# Two New Seco-Labdane Diterpenoids from the Leaves of *Callicarpa nudiflora*

**DOI:** 10.3390/molecules27134018

**Published:** 2022-06-22

**Authors:** Xia Guo, Yao Zhang, Yin Xiao, Lu Zhou, Shaoyang Yin, Xifeng Sheng, Hongling Xiang, Hui Zou

**Affiliations:** 1 Key Laboratory of Study and Discovery of Small Targeted Molecules of Hunan Province, School of Medicine, Hunan Normal University, Changsha 410013, China; 20192019912@hunnu.edu.cn (X.G.); 20192019914@hunnu.edu.cn (Y.Z.); 202120193372@hunnu.edu.cn (L.Z.); 201830192049@hunnu.edu.cn (S.Y.); 202020191550@hunnu.edu.cn (X.S.); 202020191549@hunnu.edu.cn (H.X.); 2 Department of Pharmacy, Central South University Xiangya School of Medicine Affiliated Haikou Hospital, Haikou 570208, China; xxyy_614@163.com

**Keywords:** *Callicarpa nudiflora*, diterpenoids, seco-labdane diterpenoids, NO inhibitory effects

## Abstract

Two new seco-labdane diterpenoids, nudiflopene N (1) and nudiflopene O (2), and four known compounds were isolated from the leaves of *Callicarpa nudiflora*. The structures of the new compounds were established by 1D-, 2D-NMR, and HR-ESI-MS spectral analyses. Compounds **1**–**3** showed inhibitory activities on lipopolysaccharide-induced nitric oxide (NO) production in RAW264.7 cells, and new compounds **1**–**2** exhibited more potent inhibitory activity than compound **3**. The cytotoxicity of compounds **1**–**3** against human hepatocellular carcinoma HepG2 cells and human gastric carcinoma SGC-7901 cells were evaluated, while all of them exhibited no cytotoxicity.

## 1. Introduction

The genus *Callicarpa* comprises about 190 species and is widely dispersed across tropical and subtropical Asia and Oceania. *Callicarpa nudiflora* Hook. & Arn. (Verbenaceae) is a shrub or small tree mainly found in Guangdong, Guangxi, Hainan, China, with the medicinal materials in Wuzhishan, Hainan Province representing the best specimens [1]. *C. nudiflora* is a traditional Chinese medicinal herb for eliminating stasis in order to subdue swelling and hemostasis. In the Li nationality, *C. nudiflora* is called “Bu fa” and is usually used to treat traumatic bleeding in Hainan [2]. The roots and leaves of *C. nudiflora* can be used as medicine with the effects of antifungal, antibacterial, pro-coagulation, anti-inflammatory, detoxification, blood circulation, swelling, and evacuation of wind; it can also be used to treat inflammation induced by pyogenic bacteria and acute infectious hepatitis [3,4,5]. The leaves of *C. nudiflora* are used medicinally to stop bleeding, relieve pain, dispel blood stasis, and reduce swelling [6,7,8]. Phytochemical studies have shown that the main chemical constituents of *C. nudiflora* are flavonoids, terpenoids, and lignans [9,10,11,12]. Diterpenoids are the characteristic compounds of *C. nudiflora*, and some of them showed anti-inflammatory activities by inhibiting NO production [13]. In the present study, we reported the isolation and structural elucidation of two new seco-labdane diterpenoids along with four known compounds. In addition, compounds **1**–**3** were evaluated for their anti-inflammatory activities and cytotoxicity.

## 2. Results and Discussion

### 2.1. Structure Determination

**Figure 1:**
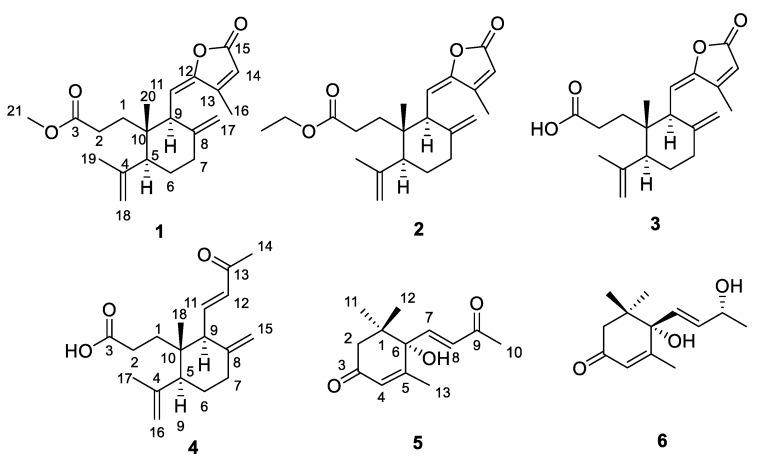
Structures of compounds **1**–**6** isolated from *Callicarpa nudiflora*.

The phytochemical study resulted in the purification of two new seco-labdane diterpenoids (1–2), two known seco-labdane diterpenoids (3–4) and two known megastigmane (**5**–**6**) from the leaves of *C. nudiflora* (Figure 1). The structures of the new compounds were elucidated through extensive spectroscopic analyses. The known compounds were identified as nudiflopene H (**3**) [14], latisilinoid (**4**) [15], (+)-dehydrovomifoliol (**5**) [16], and vomifoliol (**6**) [17] by comparing their NMR data with those reported in the literature.

**Figure 2:**
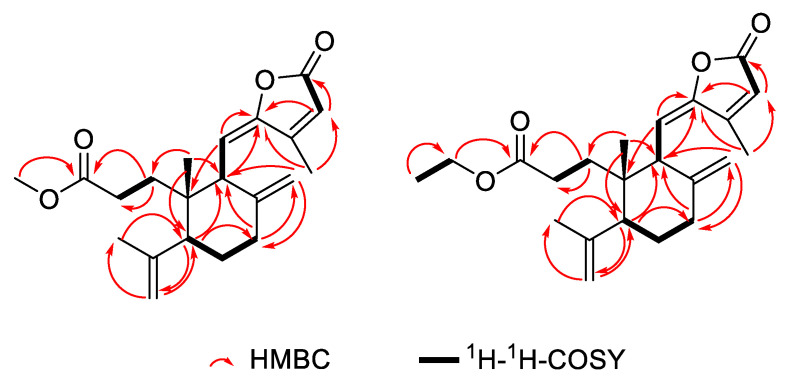
Selected HMBC and ^1^H-^1^H COSY correlations of 1 and 2.

**Figure 3:**
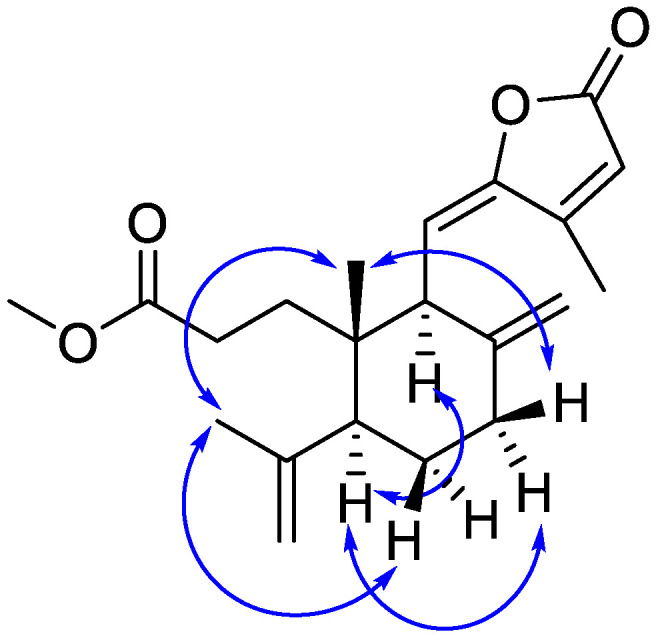
Key NOESY correlations of 1.

Compound 1 was purified as colorless oil. The molecular formula of C_21_H_28_O_4_ was analyzed from its [M + H]^+^ at 345.2058 (calcd for C_21_H_29_O_4_, 345.2066) in the HR–ESI–MS. This molecular was consistent with the ^1^H and ^13^C NMR data (Table 1). The ^1^H NMR spectrum of 1 (see Supplementary) exhibited signals for one aliphatic methyl singlet at *δ* 0.88 (3H, s, H-20), four aliphatic methylenes at *δ* 2.44 (1H, m, H-1a), 2.36 (1H, m, H-7α), 2.27 (1H, m, H-1b), 2.16 (1H, m, H-7β), 1.51 (1H, m, H-6β), 1.50 (1H, m, H-1a), 1.40 (1H, m, H-1b), 1.24 (1H, m, H-6α), six olefinic protons at *δ* 6.20 (1H, s, H-14), 5.64 (1H, d, *J* = 10.8 Hz, H-11), 4.88 (1H, s, H-18a), 4.81 (1H, s, H-17a), 4.76 (1H, s, H-18b), 4.53 (1H, s, H-17b), and one methoxy singlet at *δ* 3.50 (3H, s, H-21). The ^13^C NMR spectrum (see Supplementary) of 1 showed 21 carbon resonances including the corresponding methoxy carbon at *δ* 51.7 (C-21), two carbonyl carbon at *δ* 173.9 (C-3), 169.1 (C-15), and eight olefinic carbons at *δ* 156.2 (C-13), 151.3 (C-12), 148.1 (C-8), 146.8 (C-4), 116.3 (C-14), 114.4 (C-18), 110.6 (C-11) and 109.6 (C-17). With the aid of DEPT and HSQC spectra, the remaining ten aliphatic carbons were classified into three methyls at *δ* 23.8 (C-19), 17.1 (C-20), and 11.9 (C-16), four methylenes at *δ* 23.8 (C-19), 17.1 (C-20), and 11.9 (C-16), two methines at *δ* 49.4 (C-5), 47.2 (C-9), and one quaternary carbon at *δ* 41.6 (C-10). According to these spectroscopic data, compound **1** was inferred to be a diterpenoid carrying a methoxy group. The diterpenoid scaffold was elucidated by ^1^H-^1^H COSY and HMBC spectra (Figure 2). In the HMBC spectrum, the correlations were observed for H_3_-20 to C-5, C-9, and C-10, H_2_-17 to C-7, C-8, and C-9, and H-5 to C-4, C-6, C-7, C-9, C-10, C-18, C-19 and C-20, H_2_-18 to C-19 and C-5; H_3_-19 to C-5 and C-18, together with the ^1^H-^1^H COSY correlations, indicated the presence of a six-membered ring with a methyl group (20-Me) and an isopropenyl group attached at C-5. The residual moiety was extrapolated to form a five-membered unsaturated lactone ring with a methyl group attached at C-13, which was confirmed by the HMBC couplings of H_3_-16 to C-12, C-13, and C-14, and H-14 to C-12, C-13, C-15, and C-16. This five-membered unsaturated lactone ring was linked to C-9 of the six-membered ring via the olefinic carbon C-11 (*δ* 110.6), confirmed by the HMBC correlations of H-11 to C-8, C-9, C-10, C-12, and C-13. All of the above analysis permitted the planar structure of 1 to be elucidated. The configuration of compound **1** was elucidated based on the NOESY spectrum and compared the optical rotation and electronic circular dichroism (ECD) spectrum (see Supplementary) with the known nudiflopene H [14]. The NOESY spectrum (Figure 3) showed the crosspeaks for H-5/H-9, H-5/H-7α, H_3_-20/H-19, and H_3_-20/H-6β, which suggested the relative configuration of 1. The ECD spectrum of 1 showed a negative cotton effect, which was consistent with nudiflopene H, so the absolute configuration of 1 was assigned to be 5*S*, 9*S*, and10*S*. Therefore, the structure of compound **1** was elucidated and named nudiflopene N.

Compound **2** was purified as colorless oil. The molecular formula C_22_H_30_O_4_ was analyzed from its [M + H]^+^ at 359.2224 (calcd for C_22_H_31_O_4_, 359.2222) in the HR–ESI–MS spectrum. This molecular was consistent with the ^1^H and ^13^C NMR data (Table 1). The ^1^H and ^13^C NMR spectra (see Supplementary) of 2 showed high similarity to those of 1, implying a structurally similar diterpenoid for 2. According to their NMR data, the signals for the methoxy group in compound **1** were replaced by the ethoxy group in 2, which was supported by the DEPT experiments and 2D NMR spectra. The further interpretation of 2D NMR data led to the assignments of all the proton and carbon signals. The configuration of 2 was also identical with compound **1** based on the NOESY spectrum, optical rotation data, and ECD spectrum (see Supplementary). Therefore, the structure of compound **2** was elucidated and gave a successive name nudiflopene O.

Compounds **1** and **2** are derivatives of nudiflopene H (3). To prove that compounds **1** and **2** are not artifacts, we extracted the medicinal materials again with ethyl acetate and analyzed its constituents with HPLC-DAD. The results showed that compounds **1** and **2** can be found in the HPLC chromatogram of the ethyl acetate extract based on the retention time and UV spectrum (see Supplementary). Therefore, compounds **1** and **2** are not artifacts.

### 2.2. NO Inhibitory Activities

**Figure 4:**
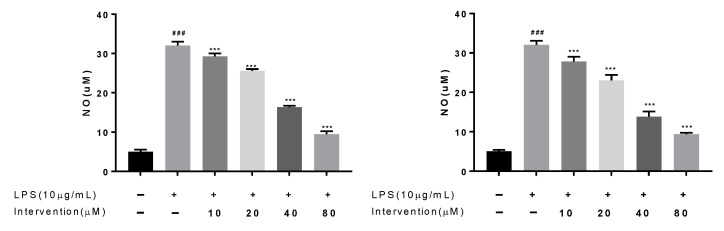
The NO inhibitory activity of compounds **1** and **2** in LPS-activated RAW264.7 cells. ### *p* < 0.001 vs. control group, *** *p* < 0.001 vs. LPS group (*n* = 3).

NO was considered as a key inflammatory mediator that may be helpful to treat inflammation. RAW264.7 is derived from Abelson murine leukemia virus–induced tumor cells, which have a strong ability to phagocytize antigens and play key roles in inflammatory, immune, and phagocytic responses. At present, the anti-inflammatory activity of natural products using LPS-induced RAW264.7 macrophages as screening model has been widely used. Some research evidenced that such diterpenoids showed NO inhibitory activity, so compounds **1**–**3** were assayed for their NO inhibitory effects in RAW 264.7 cells. Compounds **1**–**3** inhibited LPS-induced NO production in RAW 264.7 cells with IC_50_ values of 34.43 ± 1.37, 29.87 ± 2.50, and 66.58 ± 2.63 µM, respectively (Figure 4).

### 2.3. Cytotoxic Effects against Cancer Cells

Compounds 1–3 were evaluated for their cytotoxicity against HepG2 and SGC-7901 cells. All of them had no activity against these two cell lines.

## 3. Materials and Methods

### 3.1. General Experimental Procedures

Optical rotations were recorded on a Bellingham-Stanley ADP 440+ polarimeter (Bellingham-Stanley Ltd., Tunbridge Wells, UK). ECD spectra were obtained on a JASCO J-715 CD spectrometer (JASCO Corporation, Tokyo, Japan). ^1^H NMR, ^13^C NMR, Distortionless Enhancement by Polarization Transfer (DEPT), ^1^H-^1^H Correlated Spectroscopy (^1^H-^1^H COSY), Heteronuclear Multiple Quantum Correlation (HMQC), and Heteronuclear Multiple Bond Correlation (HMBC) experiments were performed on a Bruker Avance Neo 400 MHz NMR spectrometer (Billerica, MA, USA); TMS was used as international standard. Mass spectra were obtained on a Bruker micro TOF mass spectrometer (ESI-MS) (Billerica, MA, USA). High-performance liquid chromatography (HPLC) was performed using an Agilent 1260 Series HPLC system (Agilent Technologies Inc., Santa Clara, CA, USA) equipped with a four-pump with an in-line degasser, autosampler, oven, and Diode-array detector (DAD). The semi-preparative HPLC was performed using a YMC ODS-A chromatographic column (250 × 10 mm, μm).

### 3.2. Plant Material

The air-dried leaves of *Callicarpa nudiflora* were collected from Wuzhishan of Hainan Province, China in October 2015 and identified by Prof. Xi-Feng Sheng (Hunan Normal University, China). A voucher specimen (No. LHZZ-2015) has been deposited in the Department of Pharmacy, School of Medicine, Hunan Normal University.

### 3.3. Extraction and Isolation

The air-dried leaves of *C. nudiflora* (8.5 kg) were extracted twice with EtOH-H_2_O (80:20, *v*/*v*), and the concentrated liquid was dispersed in water and using petroleum ether, ethyl acetate, and *n*-butanol to extract twice to obtain three portions of petroleum ether (170 g), ethyl acetate (258 g), and *n*-butanol. The ethyl acetate portion (230 g) was subjected on a silica gel column with a gradient elution of CH_2_Cl_2_-MeOH (100:0-0:100) to obtain 9 fractions (A–I). Based on TLC analysis, the fractions B-D were combined and subjected on a silica gel column chromatography using CH_2_Cl_2_-MeOH (100:0-0:100) as a gradient elution to obtain 200 fractions. Fr. 47–51 was purified by semi-preparative HPLC (3.0 mL/min, 254 nm) with ACN–H_2_O (65:35, *v*/*v*) to obtain compound **6** (t_R_ = 34.3 min, 9.0 mg). Fr. 20–40 were combined and chromatographed on an ODS column chromatography to obtain 200 fractions by a gradient elution of MeOH-H_2_O (40:60–100:0). Fr. 27–31 was purified by semi-preparative HPLC (3.0 mL/min, 254 nm) with ACN–H_2_O (14:86, *v*/*v*) to obtain compound **5** (t_R_ = 34.0 min, 4.2 mg). Fr. 79–120 was purified by semi-preparative HPLC (3.0 mL/min, 254 nm) with ACN–H_2_O (75:25, *v*/*v*) to obtain compound **1** (t_R_ = 42.7 min, 6.2 mg). The petroleum ether portion (161 g) was subjected on a silica gel column with a gradient elution of PE-EA (10:0.6-0:1) to obtain 226 fractions. Fr. 47–51 was purified by semi-preparative HPLC (3.0 mL/min, 254 nm) with ACN–H_2_O (74:26, *v*/*v*) to obtain compound 2 (t_R_ = 37.0 min, 8.5 mg). Fr. 111–112 was purified by semi-preparative HPLC (3.0 mL/min, 254 nm) with ACN–H_2_O (51:49, *v*/*v*) to obtain compound **4** (t_R_ = 25.6 min, 9.5 mg). Fr. 120–123 was purified by semi-preparative HPLC (3.0 mL/min, 254 nm) with ACN–H_2_O (47:53, *v*/*v*) to obtain compound **3** (t_R_ = 37.2 min, 12.0 mg).

Nudiflopene N (**1**): Colorless oil. [α]${}_{D}^{20}$ -53 (c 0.1, MeOH); ECD (MeOH): 268 (∆*ε*-2.93) nm; UV (MeOH) λmax: 274 nm; ^1^H-NMR and ^13^C-NMR (DMSO-*d*_6_) see Table 1; HR-ESI-MS calcd for C_21_H_29_O_4_ [M + H]^+^ 345.2066; found 345.2058.

Nudiflopene O (**2**): Colorless oil. [α]${}_{D}^{20}$ -65 (c 0.1, MeOH); ECD (MeOH): 269 (∆*ε* -2.17) nm; UV (MeOH) λmax: 274 nm. ^1^H-NMR and ^13^C-NMR (DMSO-*d*_6_) see Table 1; HR-ESI-MS calcd for C_22_H_31_O_4_ [M + H]^+^ 359.2222; found 359.2224.

Nudiflopene H (**3**): Colorless oil. [α]${}_{D}^{20}$ -47 (c 0.1, MeOH); UV (MeOH) λmax: 274 nm. ^1^H NMR (DMSO-*d*_6_, 400 MHz): *δ*_H_ 6.18 (1H, s, H-14), 5.64 (1H, d, *J* = 10.6 Hz, H-11), 4.87 (1H, s, H-18), 4.79 (1H, s, H-17), 4.73 (1H, s, H-18), 4.50 (1H, s, H-17), 3.21 (1H, d, *J* = 10.5 Hz, H-9), 2.37 (1H, d, *J* = 13.2 Hz, H-7), 2.37 (1H, m, H-5), 2.26 (1H, s, H-2), 2.23 (3H, s, H-16), 2.14 (1H, m, H-7), 2.06 (1H, s, H-2), 1.74 (3H, s, H-19), 1.73 (1H, m, H-6), 1.50 (1H, m, H-6), 1.47 (1H, m, H-1), 1.32 (1H, m, H-1), 0.87 (3H, s, H-20). ^13^C NMR (DMSO-*d*_6_, 100 MHz): *δ*_C_ 169.2 (C-3), 169.1 (C-15), 156.1 (C-13), 151.2 (C-12), 148.4 (C-8), 147.0 (C-4), 116.3 (C-14), 114.1 (C-18), 111.0 (C-11), 109.4 (C-17), 49.3 (C-5), 47.5 (C-9), 41.4 (C-10), 35.9 (C-7), 34.9 (C-1), 29.2 (C-2, 6),24.4 (C-19), 17.1 (C-20), 12.0 (C-16).

Latisilinoid (**4**): White amorphous powder. UV λmax: 196, 226 nm. ^1^H NMR (DMSO-*d*_6_, 400 MHz): *δ*_H_ 6.90 (1H, dd, *J* = 15.9, 10.3 Hz, H-11), 6.12 (1H, d, *J* = 15.9 Hz, H-12), 4.88 (1H, s, H-16), 4.80 (1H, s, H-15), 4.72 (1H, s, H-16), 4.42 (1H, s, H-15), 2.84 (1H, d, *J* = 10.3 Hz, H-9), 2.31 (2H, s, H-2, 5), 2.26 (4H, s, H-7, 14), 2.15 (1H, m, H-7), 2.08 (1H, m, H-2), 1.73 (4H, s, H-2, 17), 1.49 (2H, m, H-1, 6), 1.27 (1H, m, H-1), 0.86 (3H, s, H-18). ^13^C NMR (DMSO-*d*_6_, 100 MHz): *δ*_C_ 198.5 (C-13), 175.9 (C-3), 148.9 (C-8), 146.3 (C-4, 11), 133.9 (C-12), 114.2 (C-16), 109.5 (C-15), 52.9 (C-9), 49.1 (C-5), 40.9 (C-10), 35.9 (C-2), 34.3 (C-1), 29.1 (C-6), 28.3 (C-7), 27.3 (C-14), 24.3 (C-17), 17.4 (C-18).

(+)-dehydrovomifoliol (**5**): Yellow amorphous powder. UV λmax: 240 nm. ^1^H NMR (DMSO-*d*_6_, 400 MHz): *δ*_H_ 7.02 (1H, d, *J* = 15.8 Hz, H-7), 6.45 (1H, d, *J* = 15.8 Hz, H-8), 5.96 (1H, s, H-4), 2.63 (1H, d, *J* = 17.0 Hz, H-2), 2.27 (1H, s, H-2), 2.33 (3H, s, H-10), 1.92 (3H, d, *J* = 1.4 Hz, H-13), 1.08 (3H, s, H-11), 1.04 (3H, s, H-12). ^13^C NMR (DMSO-*d*_6_, 100 MHz): *δ*_C_ 199.4 (C-9), 199.0 (C-3), 163.2 (C-5), 146.9 (C-7), 130.4 (C-8), 126.6 (C-4), 78.5 (C-6), 49.1 (C-2), 41.2 (C-1), 26.2 (C-10), 23.3 (C-12), 22.1 (C-11), 17.7 (C-13).

Vomifoliol (**6**): Yellow amorphous powder. UV λmax: 196, 242 nm. ^1^H NMR (DMSO-*d*_6_, 400 MHz): *δ*_H_ 5.79 (1H, s, H-4), 5.69 (1H, s, H-8), 5.67 (1H, d, *J* = 3.2 Hz, H-7), 4.19 (1H, m, H-9), 2.37 (1H, d, *J* = 16.7 Hz, H-2), 2.06 (1H, d, *J* = 16.7 Hz, H-2), 1.81 (3H, d, *J* = 1.2 Hz, H-13), 1.12 (3H, d, *J* = 6.4 Hz, H-10), 0.93 (3H, s, H-11), 0.92 (3H, s, H-12). ^13^C NMR (DMSO-*d*_6_, 100 MHz): *δ*_C_ 197.8 (C-3), 164.8 (C-5), 136.3 (C-7), 128.3 (C-8), 125.9 (C-4), 78.2 (C-6), 66.5 (C-9), 49.8 (C-2), 41.3 (C-1), 24.5 (C-12), 24.4 (C-10), 23.5 (C-11), 19.4 (C-13).

### 3.4. Bioassay for NO Inhibitory Activities

NO is an important inflammatory factor, and the NO inhibitory effects of compounds **1**–**3** were examined by inhibiting NO release in LPS-induced murine macrophage RAW 264.7 cells. The cells were cultured in a cell temperature incubator and a DMEM at 37 °C in 5% CO_2_. The cells were seeded in 96-well culture plates (10,000 cells/well) and allowed to adhere for 24 h at 37 °C. At the same time, a blank control group and a drug group were set up and cultured 2 h in a cell temperature incubator. 10 μg/mL of LPS (Sigma Chemical Co., St. Louis, MI, USA) per well were added to induce inflammation, and the culture was continued in the incubator for 24 h. As a parameter of NO synthesis, the nitrite concentration was measured by the Griess reaction using the supernatant of the RAW 264.7 cells. 50 μL of cell supernatant per well, then Griess reagent I and Griess reagent II, respectively, were added. The absorbance was read with a microplate reader (Bio-Tek Instruments, Inc., Winooski, VT, USA) at 550 nm. The experiment was performed three times. SPSS 16.0 and GraphPad Prism 6.01 software were used for statistical analysis.

### 3.5. Cytotoxicity Assay

The cytotoxicity assay was carried out using the CCK-8 method. HepG2 and SGC-7901 cells were cultured in a cell temperature incubator and a DMEM at 37 °C in 5% CO_2_, respectively. The cells of the logarithmic growth phase were seeded into 96-well plates with a density of 4000 cells/well in 200 µL medium, respectively. The cells were treated with the tested compounds at various concentrations (0, 10, 20, 40 and 80 µM), with sorafenib and cisplatin as positive control. Each of three parallel holes were located, then incubated for 24 h. Subsequently, the 96-well plate was taken out, and 10 μL of CCK-8 was added to the experimental and control wells; at the same time, two other individual wells were taken as blank controls, and only 10 μL of CCK-8 in 0.1 mL of DMEM was added to each well. Then incubation under the same conditions was conducted for 4 h. The optical density (OD) was measured at 450 nm using a Bio-Tek Synergy (Bio-Tek Instruments, USA). The experiment was repeated 3 times. Finally, the impact of drugs on cell growth inhibition rate and IC_50_ values was calculated.

## 4. Conclusions

In this study, two new seco-labdane diterpenoids, nudiflopene N (1) and nudiflopene O (2), along with four known compounds, were isolated from the leaves of *Callicarpa nudiflora* Hook. & Arn. The structures of the new compounds were elucidated by spectroscopic analysis. Compounds **1**–**3** from this plant were evaluated for their NO inhibitory activity and cytotoxicity against human hepatocellular carcinoma HepG2 cells and human gastric carcinoma SGC-7901 cells. All of them showed good inhibitory activity on the LPS-induced NO production in RAW 264.7 cells, and compounds **1**–**2** showed more potent activity than compound **3**.

## Figures and Tables

**Table 1:** ^1^H (400 MHz) and ^13^C-NMR (100 MHz) spectral data of 1–2 in DMSO-*d*_6_.

	1		2	
Position	*δ*_H_ (Multiplicity, *J* in Hz)	*δ* *_C_*	*δ*_H_ (Multiplicity, *J* in Hz)	*δ* *_C_*
1a	1.50 (1H, m)	34.1	1.50 (1H, m)	34.2
1b	1.40 (1H, m)		1.38 (1H, m)	
2a	2.44 (1H, m)	27.7	2.41 (1H, m)	28.0
2b	2.27 (1H, m)		2.29 (1H, m)	
3	-	173.9	-	173.5
4	-	146.8	-	146.8
5	2.40 (1H, m)	49.4	2.39 (1H, m)	49.4
6α	1.24 (1H, m)	29.0	1.50 (1H, m)	29.0
6β	1.51 (1H, m)		1.73 (1H, m)	
7α	2.36 (1H, m)	35.7	2.34 (1H, m)	35.8
7β	2.16 (1H, m)		2.17 (1H, m)	
8	-	148.1	-	148.1
9	3.23 (1H, d, *J* = 10.8 Hz)	47.2	3.25 (1H, d, *J* = 10.8 Hz)	47.2
10	-	41.6	-	41.6
11	5.64 (1H, d, *J* = 10.8 Hz)	110.6	5.64 (1H, d, *J* = 10.8 Hz)	110.6
12	-	151.3	-	151.3
13	-	156.2	-	156.1
14	6.20 (1H, s)	116.3	6.20 (1H, s)	116.3
15	-	169.1	-	169.1
16	2.23 (3H, s)	11.9	2.23 (3H, s)	12.0
17a	4.81 (1H, s)	109.6	4.81 (1H, s)	109.9
17b	4.53 (1H, s)		4.54 (1H, s)	
18a	4.88 (1H, s)	114.4	4.88 (1H, s)	114.4
18b	4.76 (1H, s)		4.76 (1H, s)	
19	1.73 (3H, s)	23.8	1.73 (3H, s)	23.8
20	0.88 (3H, s)	17.1	0.88 (3H, s)	17.1
21	3.50 (3H, s)	51.7	3.94 (2H, q, *J* = 7.1 Hz)	60.2
22			1.09 (3H, t, *J* = 7.1 Hz)	14.4

## Data Availability

All data presented in this research are available in the article.

## Supplementary Materials

The following supporting information can be downloaded at: https://www.mdpi.com/article/10.3390/molecules27134018/s1, Figure S1: ^1^H NMR spectrum of 1 in DMSO-*d*_6_ (400 MHz); Figure S2: ^13^C NMR spectrum of 1 in DMSO-*d*_6_ (100 MHz); Figure S3: DEPT spectrum of 1 in DMSO-*d*_6_ (100 MHz); Figure S4: ^1^H-^1^H COSY spectrum of 1 in DMSO-*d*_6_; Figure S5: HSQC spectrum of 1 in DMSO-*d*_6_; Figure S6: HMBC spectrum of 1 in DMSO-*d*_6_; Figure S7: NOESY spectrum of 1 in DMSO-*d*_6_; Figure S8: HR-ESI-MS spectrum of 1; Figure S9: ECD sepctrum of 1; Figure S10: ^1^H NMR spectrum of 2 in DMSO-*d*_6_ (400 MHz); Figure S11: ^13^C NMR spectrum of 2 in DMSO-*d*_6_ (100 MHz); Figure S12: DEPT spectrum of 2 in DMSO-*d*_6_ (100 MHz); Figure S13: ^1^H-^1^H COSY spectrum of 2 in DMSO-*d*_6_; Figure S14: HSQC spectrum of 2 in DMSO-*d*_6_; Figure S15: HMBC spectrum of 2 in DMSO-*d*_6_; Figure S16: NOESY spectrum of 2 in DMSO-*d*_6_; Figure S17: HR-ESI-MS spectrum of 2; Figure S18: ECD sepctrum of 2. Figure S19: HPLC chromatogram of the ethyl acetate extract. Figure S20: The corresponding UV spectrum of 1 and 2. Figure S21: HPLC chromatogram of 1. Figure S22: The corresponding UV spectrum of 1. Figure S23: HPLC chromatogram of 2. Figure S24: The corresponding UV spectrum of 2.

## Sample Availability

Samples of the compounds are not available from the authors.
