# Supplementary material for: Two New Seco-Labdane Diterpenoids from the Leaves of Callicarpa nudiflora
Source: Molecules. 2022 Jun 22;27(13):4018. doi: 10.3390/molecules27134018 (PMC9267982; doi:10.3390/molecules27134018)
Supplement: Supplementary file 1 [file molecules-27-04018-s001.zip › molecules-1732720-Supplementary.pdf]

SUPPLEMENTARY MATERIAL

Two New Seco-labdane Diterpenoids from the Leaves of *Callicarpa nudiflora*

Xia Guo [1,†], Yao Zhang [1,†], Yin Xiao[2,†], Lu Zhou [1], Shaoyang Yin [1], Xifeng Sheng [1], Hongling Xiang [1] and Hui Zou [1,*]

[1] Key Laboratory of Study and Discovery of Small Targeted Molecules of Hunan Province, School of Medicine, Hunan Normal University, Changsha 410013, China; 20192019912@hunnu.edu.cn (X.G.); 20192019914@hunnu.edu.cn (Y.Z.); 202120193372@hunnu.edu.cn (L.Z.); 201830192049@hunnu.edu.cn (S.-Y.Y.); 202020191550@hunnu.edu.cn (X.-F.S.); 202020191549@hunnu.edu.cn (H.-L.X.)

[2] Central South University Xiangya School of Medicine Affiliated Haikou Hospital, Haikou, 570208, R. P. China; xxyy_614@163.com (Y.X.)

* Correspondence: zouhui@hunnu.edu.cn (H.Z.)

† These authors contributed equally to this work.

Tables of Contents	page
**Figure S1:** ^{1}H NMR spectrum of **1** in DMSO-d_6 (400 MHz)	3
**Figure S2:** ^{13}C NMR spectrum of **1** in DMSO-d_6 (100 MHz)	4
**Figure S3:** DEPT spectrum of **1** in DMSO-d_6 (100 MHz)	5
**Figure S4:** ^{1}H-^{1}H COSY spectrum of **1** in DMSO-d_6	6
**Figure S5:** HSQC spectrum of **1** in DMSO-d_6	7
**Figure S6:** HMBC spectrum of **1** in DMSO-d_6	8
**Figure S7:** NOESY sepctrum of **1** in DMSO-d_6	9
**Figure S8:** HR-ESI-MS sepctrum of **1**	10
**Figure S9:** ECD sepctrum of **1**	11
**Figure S10:** ^{1}H NMR spectrum of **2** in DMSO-d_6 (400 MHz)	12
**Figure S11:** ^{13}C NMR spectrum of **2** in DMSO-d_6 (100 MHz)	13
**Figure S12:** DEPT spectrum of **2** in DMSO-d_6 (100 MHz)	14
**Figure S13:** ^{1}H-^{1}H COSY spectrum of **2** in DMSO-d_6	15
**Figure S14:** HSQC spectrum of **2** in DMSO-d_6	16
**Figure S15:** HMBC spectrum of **2** in DMSO-d_6	17
**Figure S16:** NOESY sepctrum of **2** in DMSO-d_6	18
**Figure S17:** HR-ESI-MS sepctrum of **2**	19

Figure S18: ECD sepctrum of **2** 20

Figure S19: The HPLC chromatogram of the ethyl acetate extract 21

Figure S20: The corresponding UV spectrum of **1** and **2** 21

Figure S21: The HPLC chromatogram of **1** 22

Figure S22: The corresponding UV spectrum of **1** 22

Figure S23: The HPLC chromatogram of **2** 23

Figure S24: The corresponding UV spectrum of **2** 23

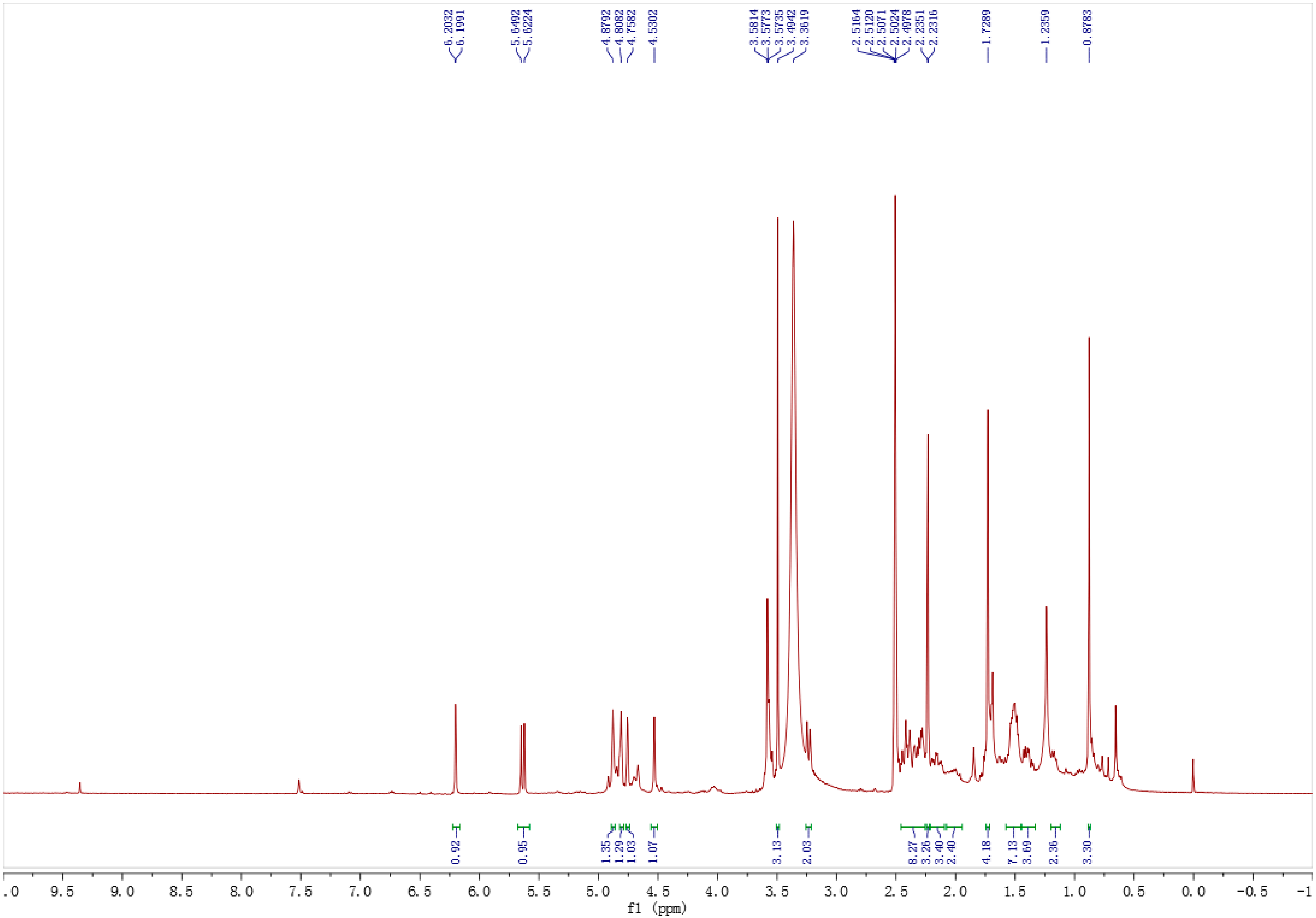

Figure S1: ^{1}H NMR spectrum of **1** in DMSO-d_6 (400 MHz).

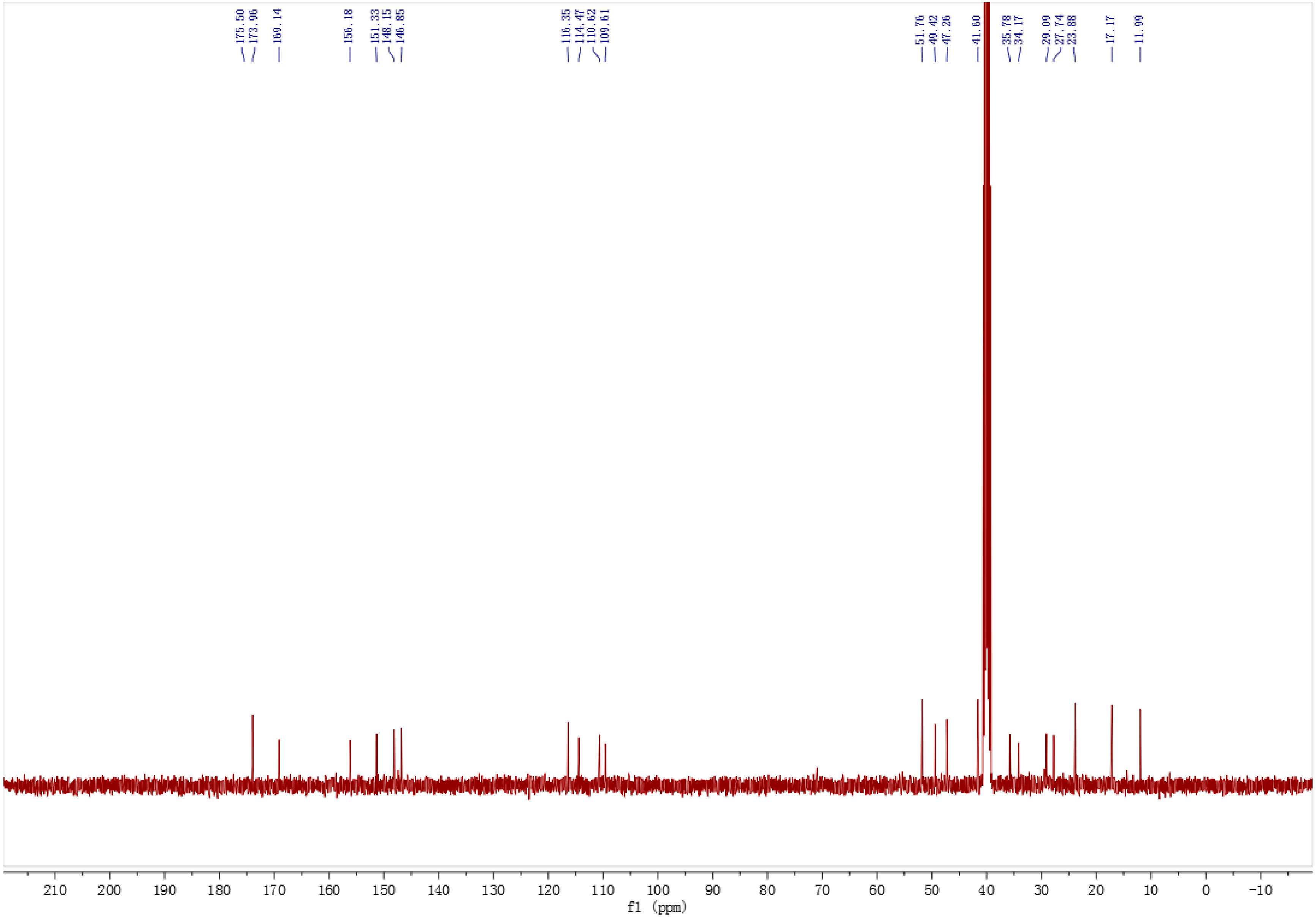

Figure S2: ^{13}C NMR spectrum of **1** in DMSO-d_6 (100 MHz).

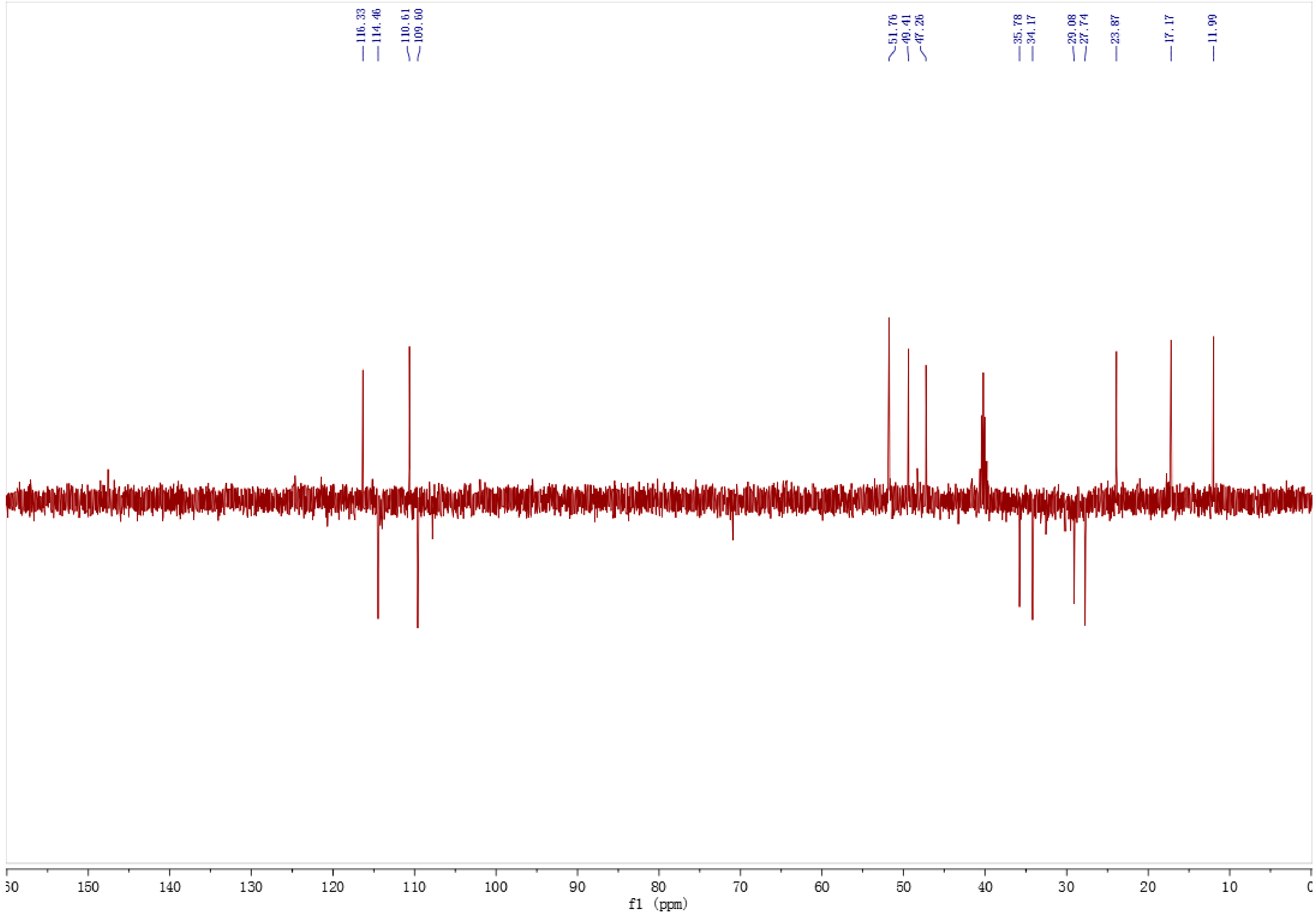

Figure S3: DEPT spectrum of **1** in DMSO-d_6 (100 MHz).

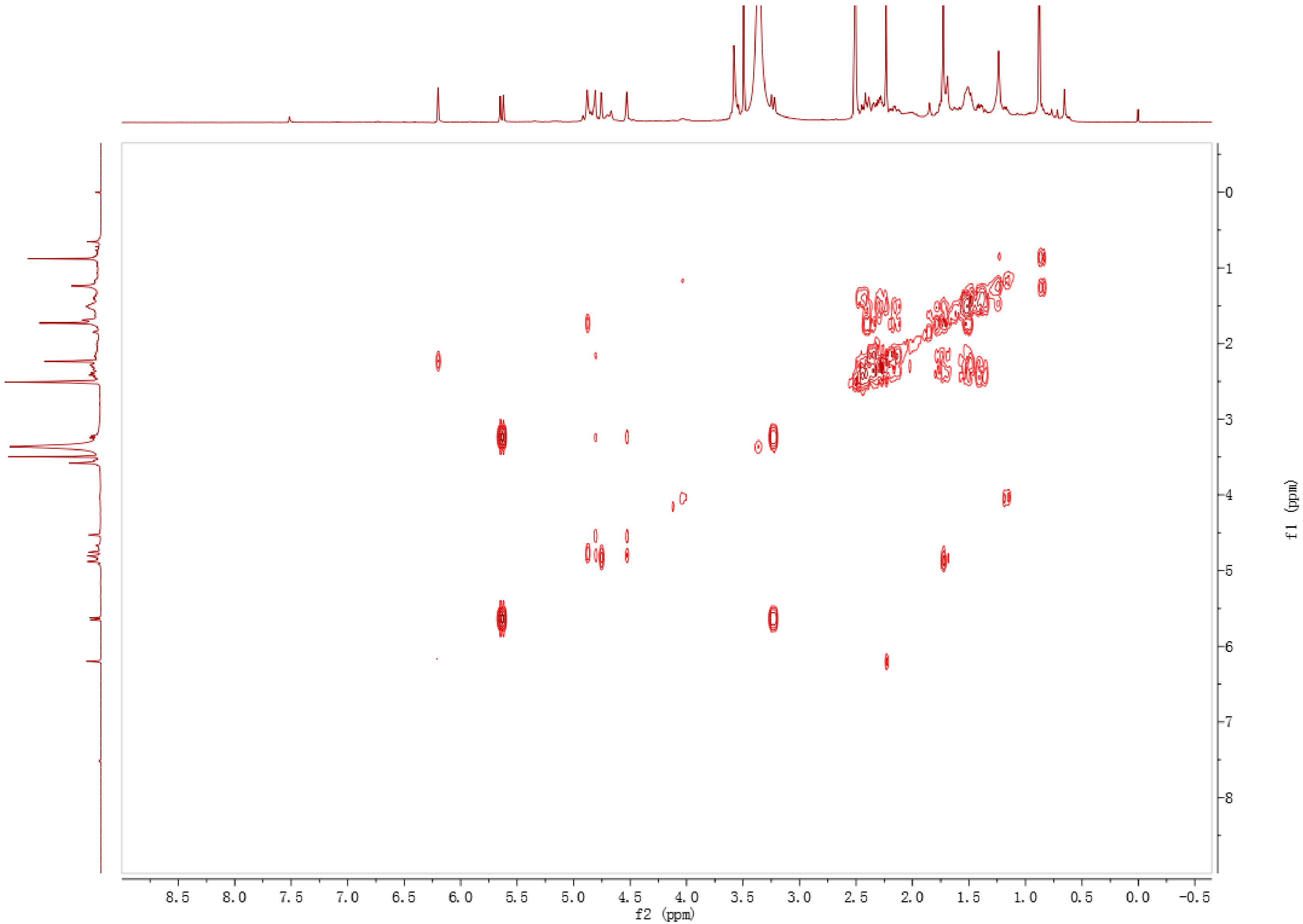

Figure S4: ^{1}H-^{1}H COSY spectrum of **1** in DMSO-d_6.

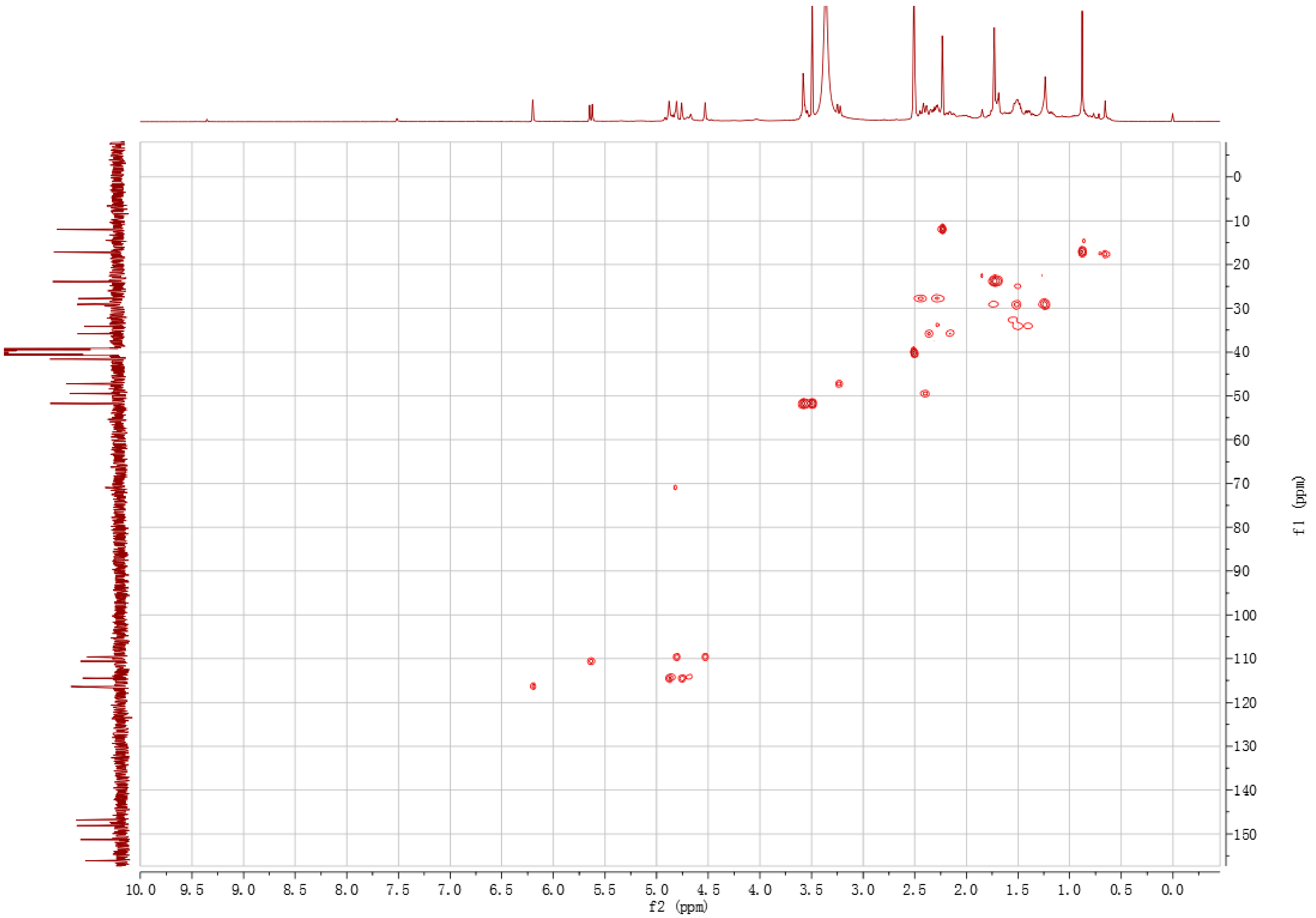

Figure S5: HSQC spectrum of compound **1** in DMSO-d_6.

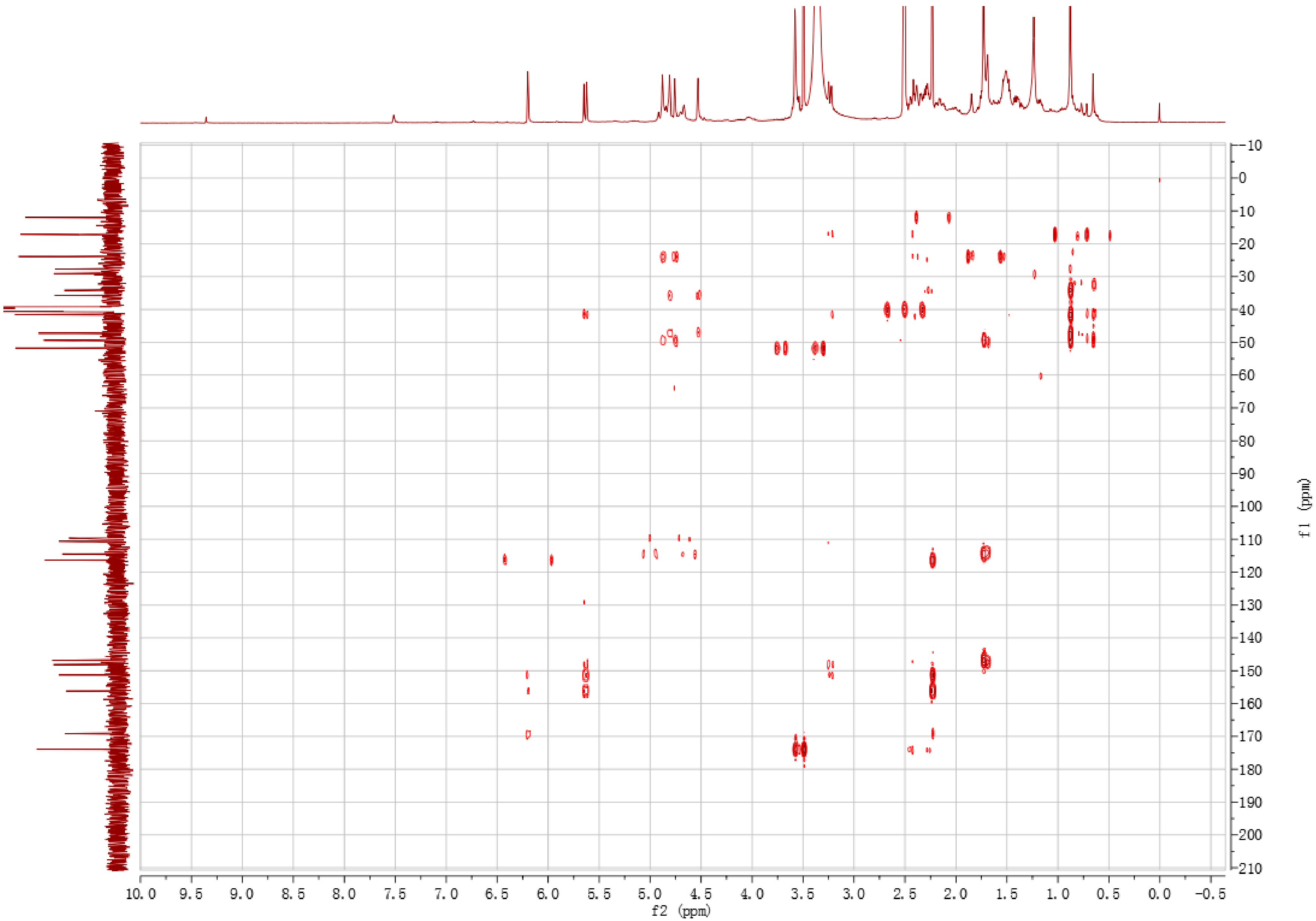

Figure S6: HMBC spectrum of **1** in DMSO-d_6.

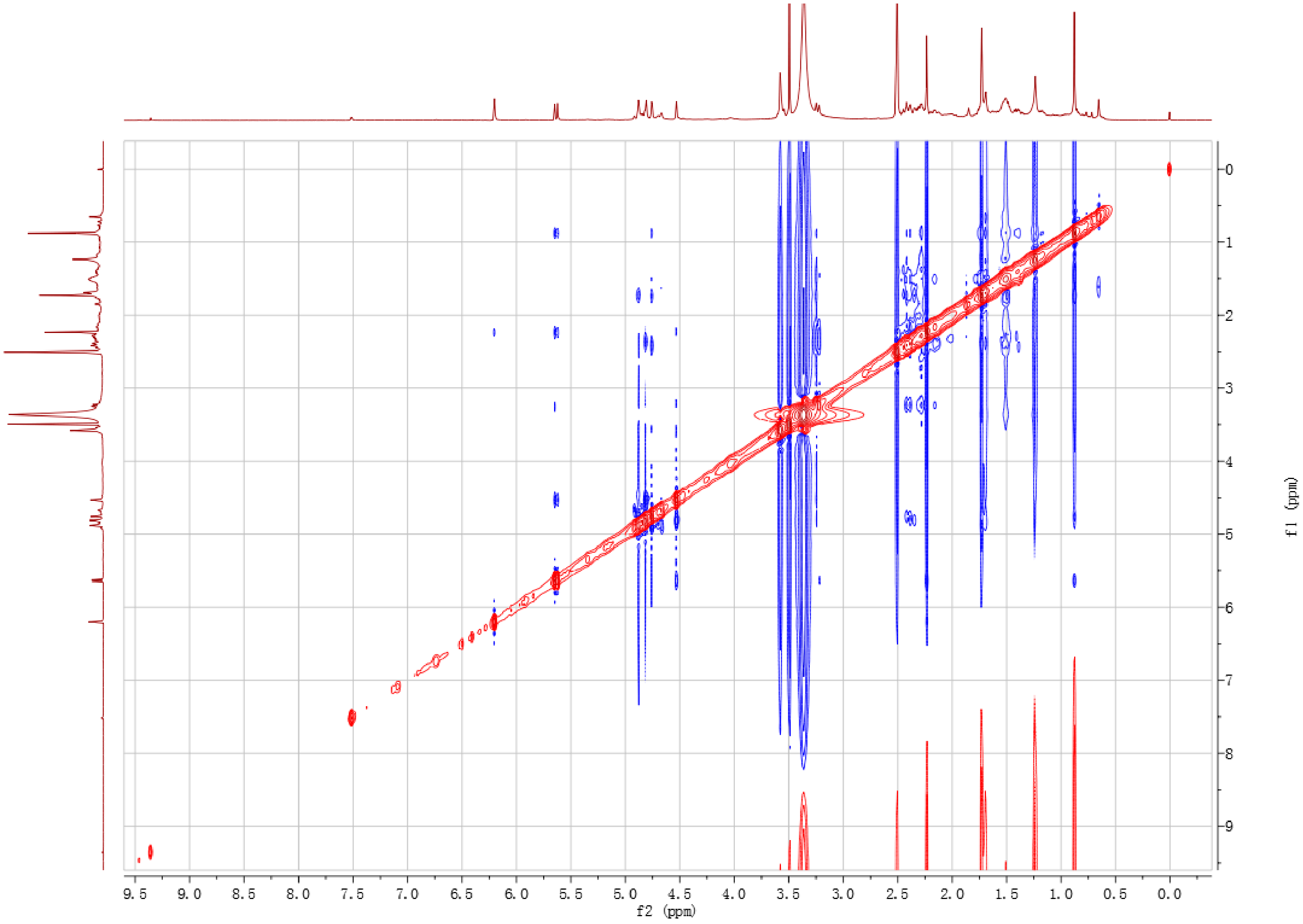

Figure S7: NOESY spectrum of **1** in DMSO-d_6.

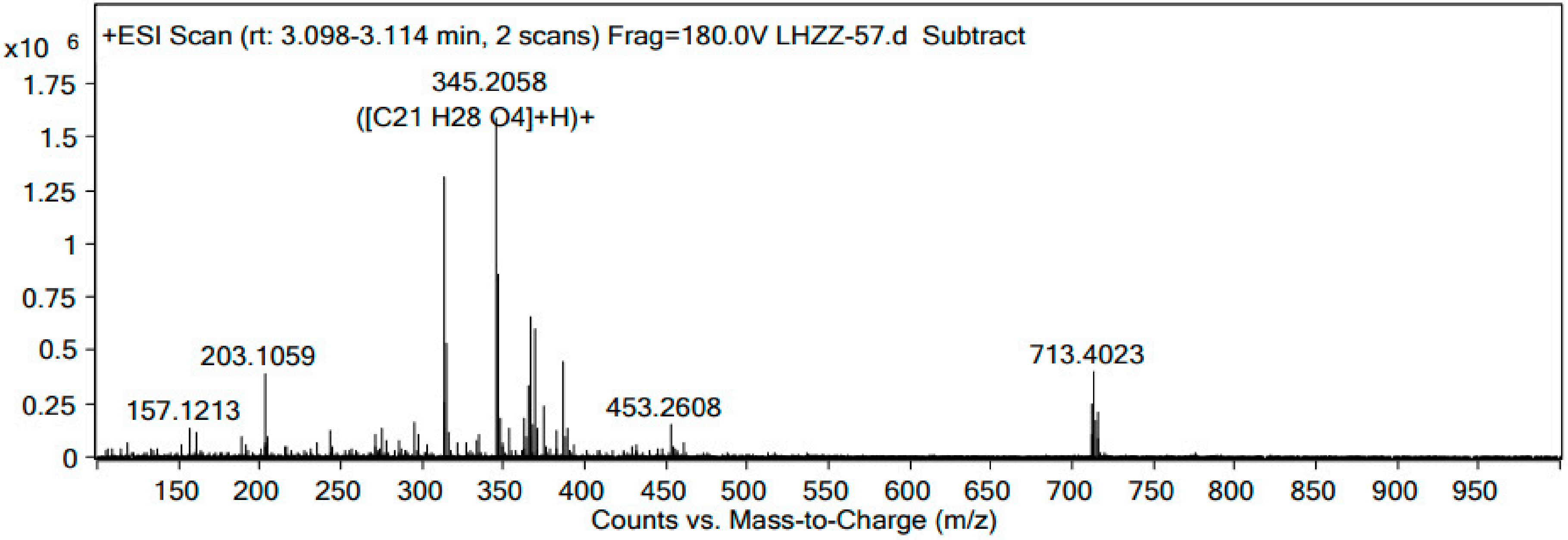

Figure S8: HR-ESI-MS spectrum of **1**.

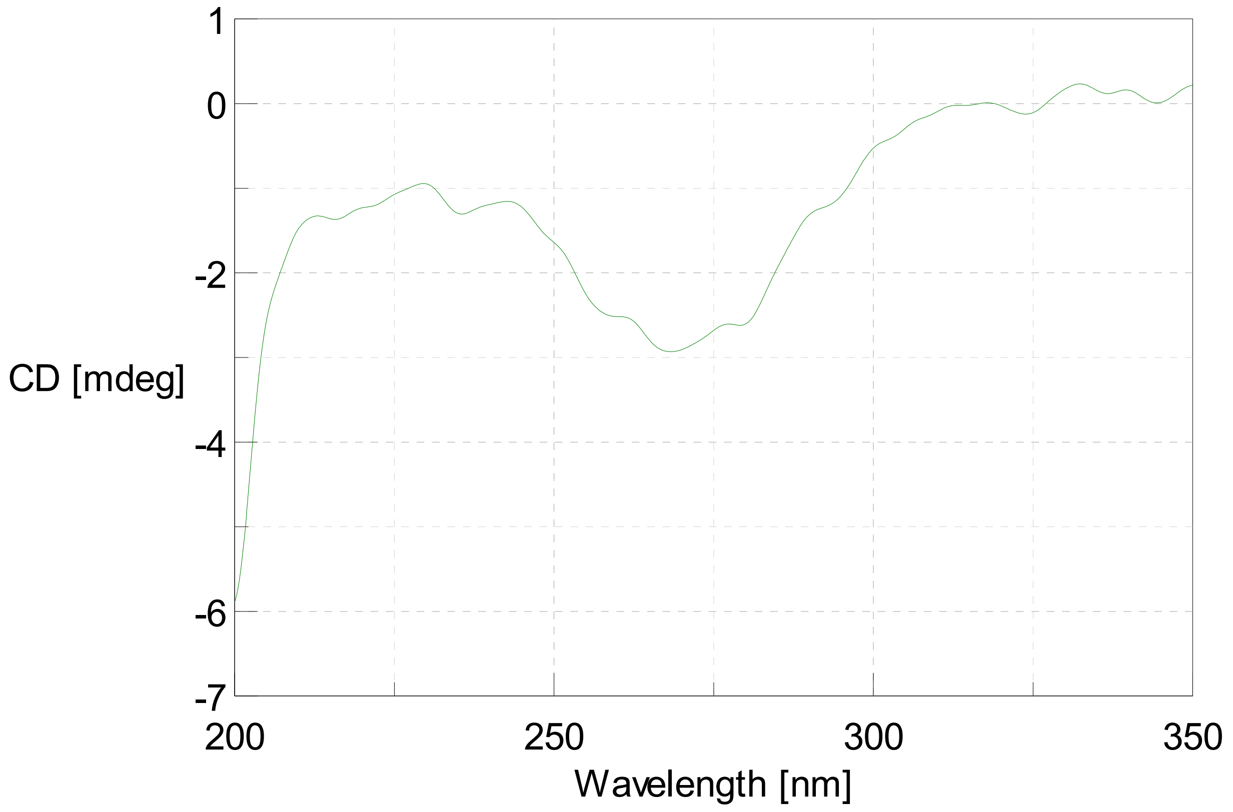

Figure S9: ECD sepctrum of **1**.

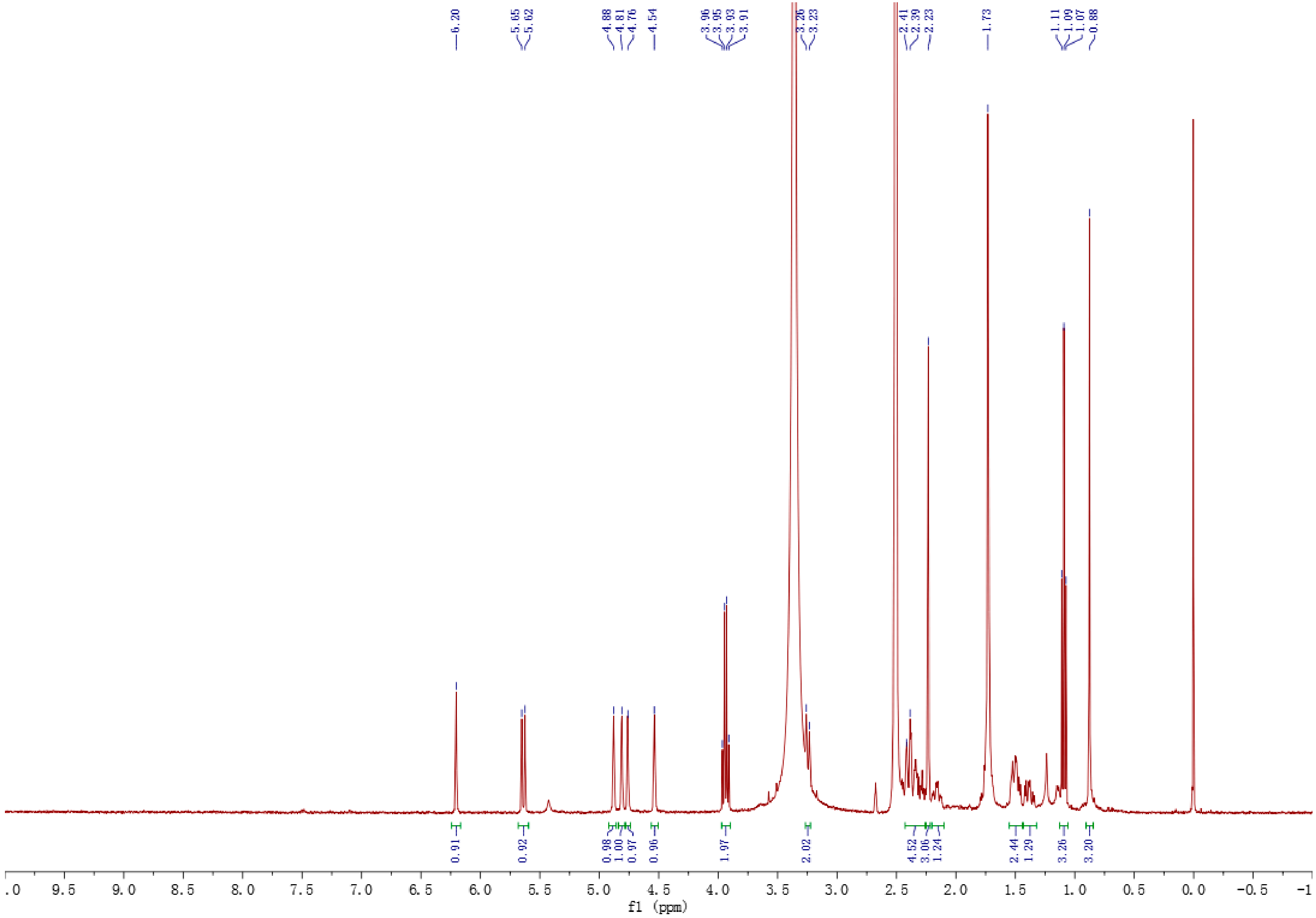

Figure S10: ^{1}H NMR spectrum of **2** in DMSO-d_6 (400 MHz).

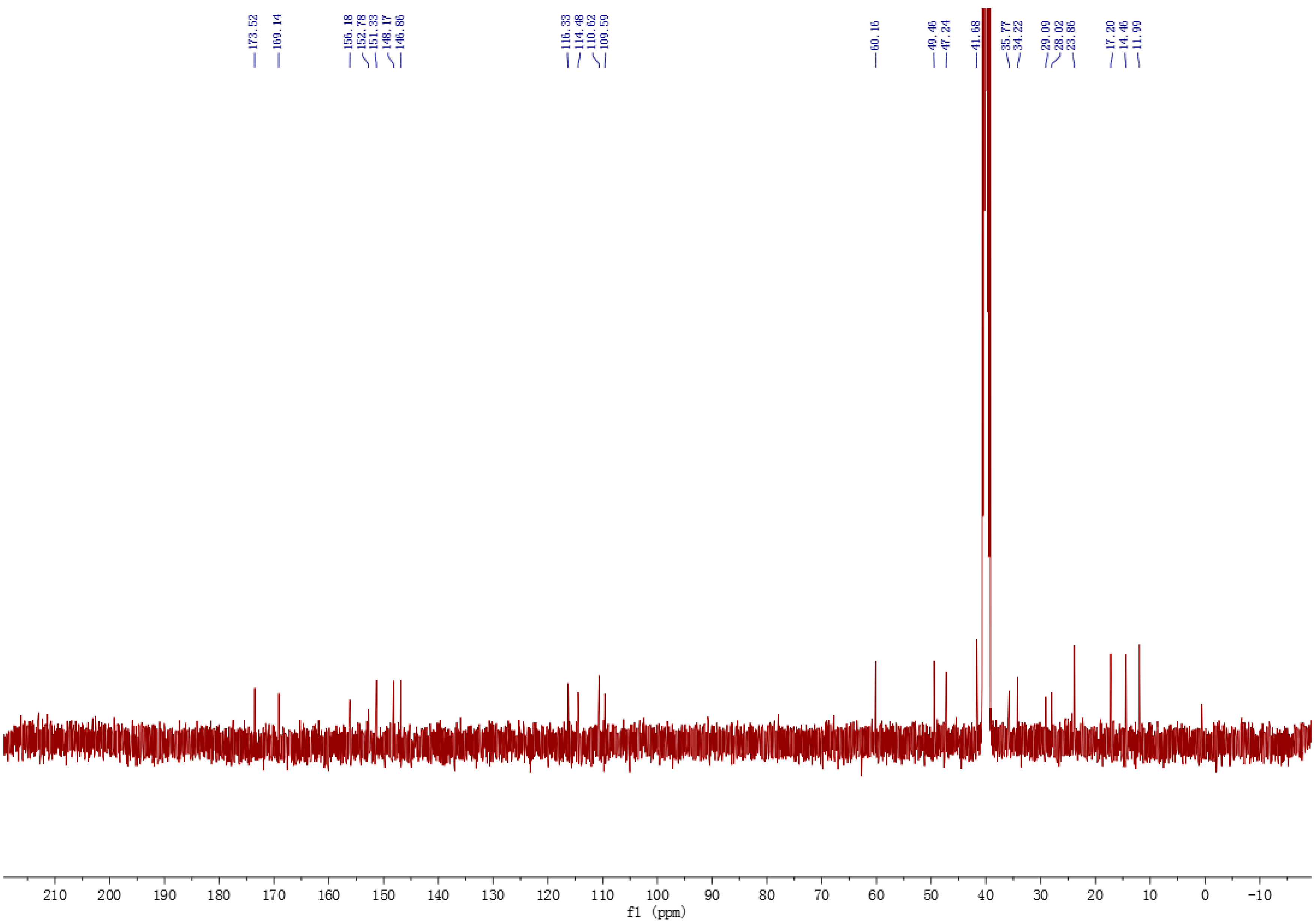

Figure S11: ^{13}C NMR spectrum of **2** in DMSO-d_6 (100 MHz).

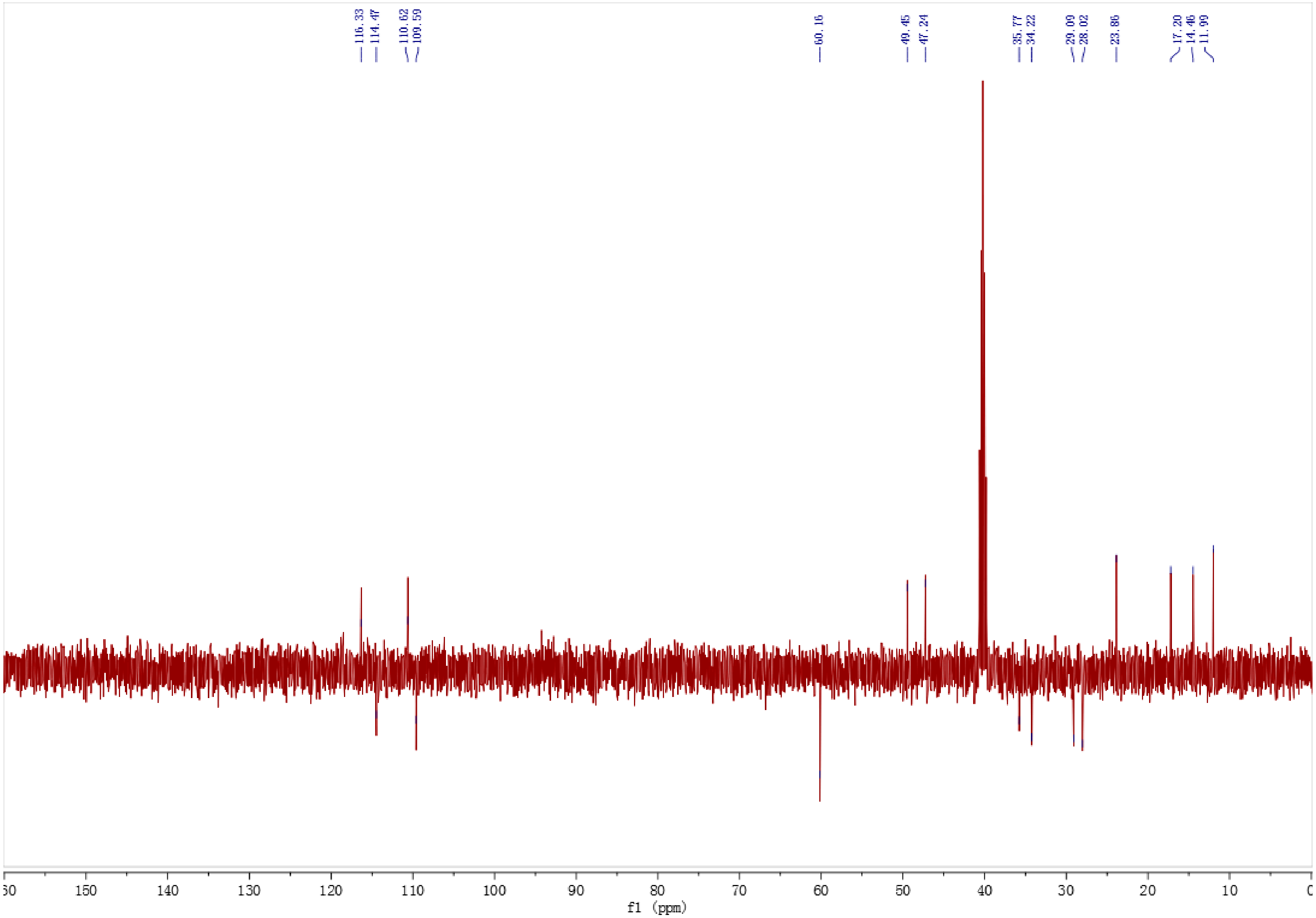

Figure S12: DEPT spectrum of **1** in DMSO-d_6 (100 MHz).

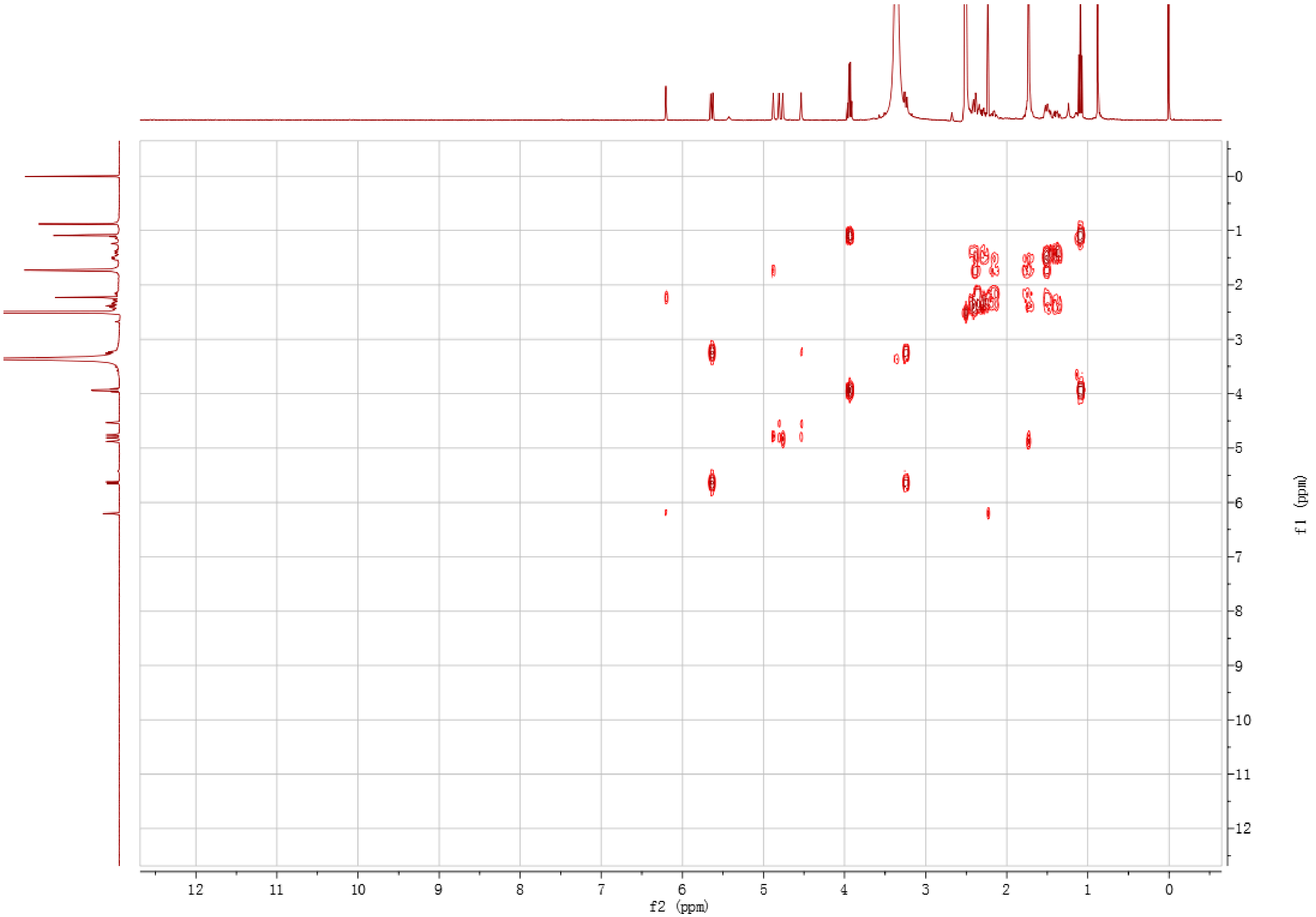

Figure S13: ^{1}H-^{1}H COSY spectrum of **2** in DMSO-d_6.

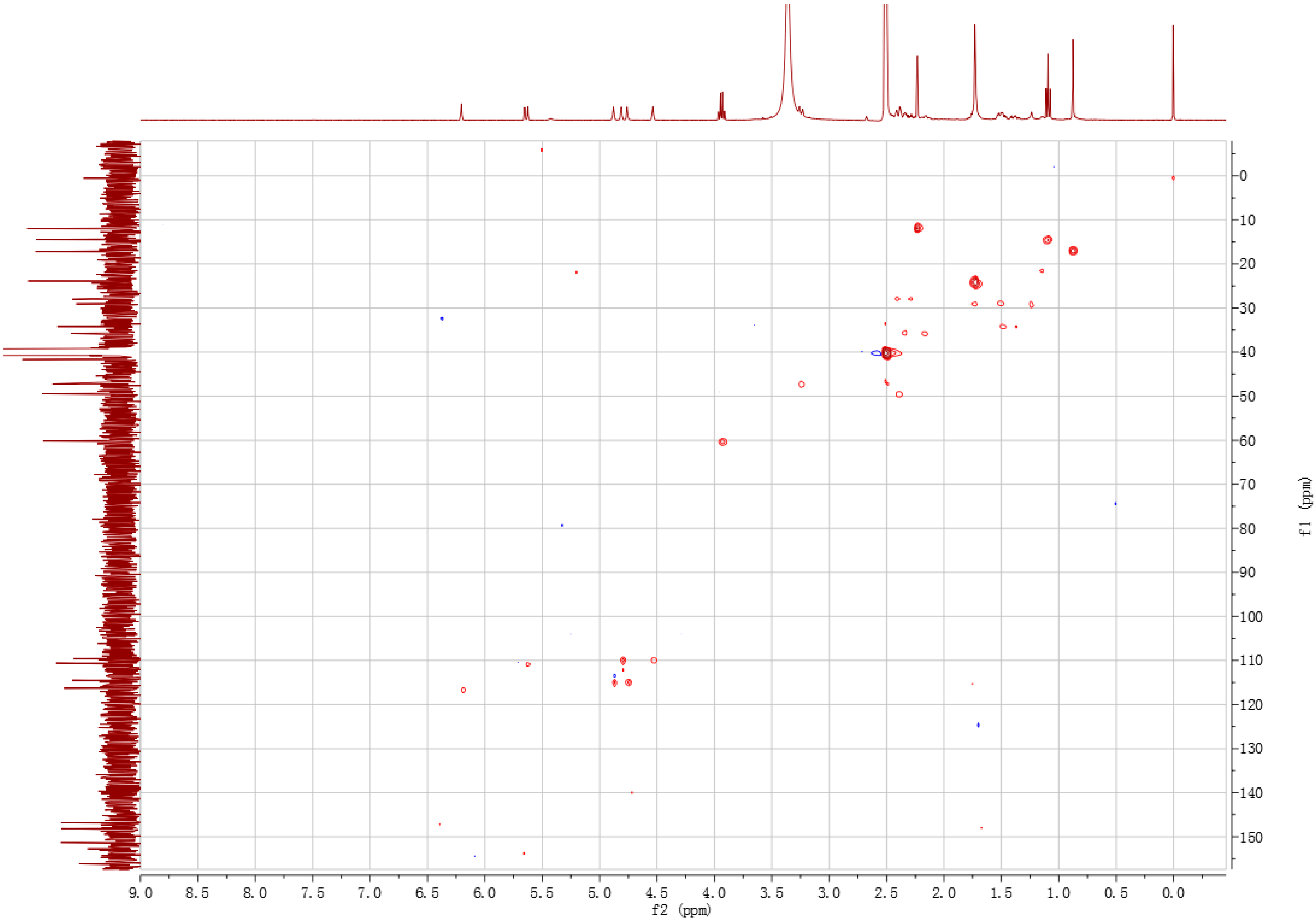

Figure S14: HSQC spectrum of compound **2** in DMSO-d_6.

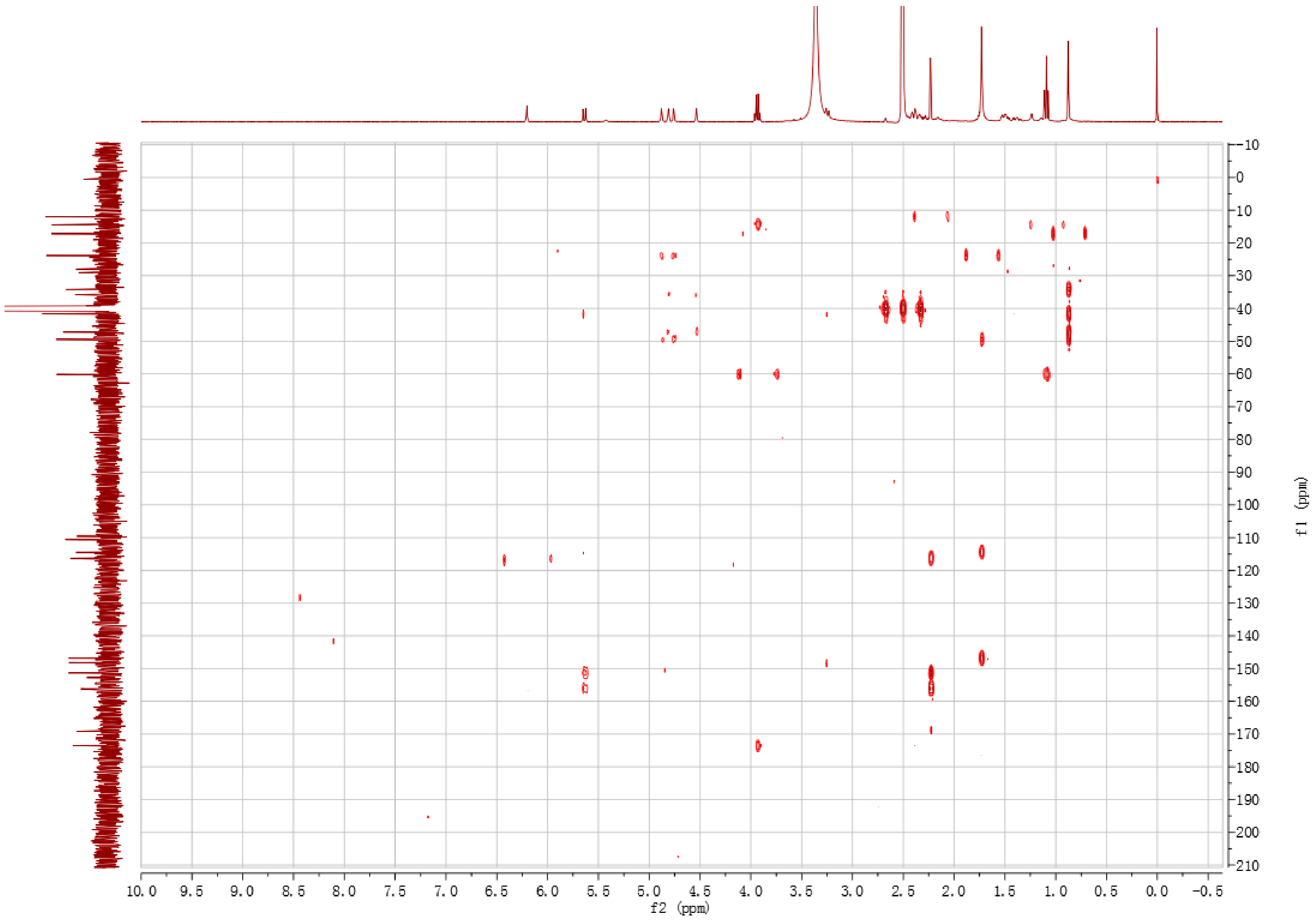

Figure S15: HMBC spectrum of compound **2** in DMSO-d_6.

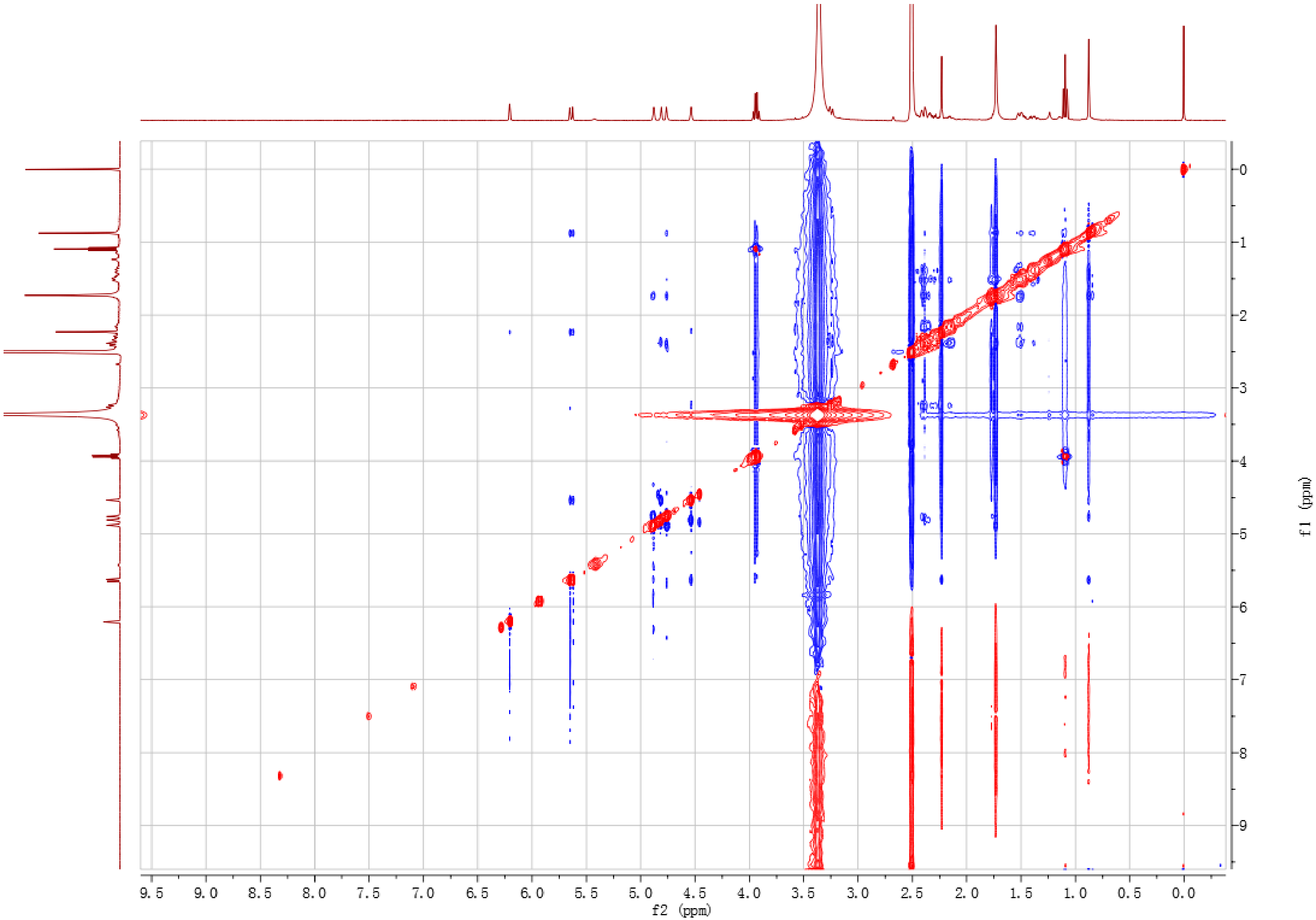

Figure S16: NOESY spectrum of **2** in DMSO-d_6.

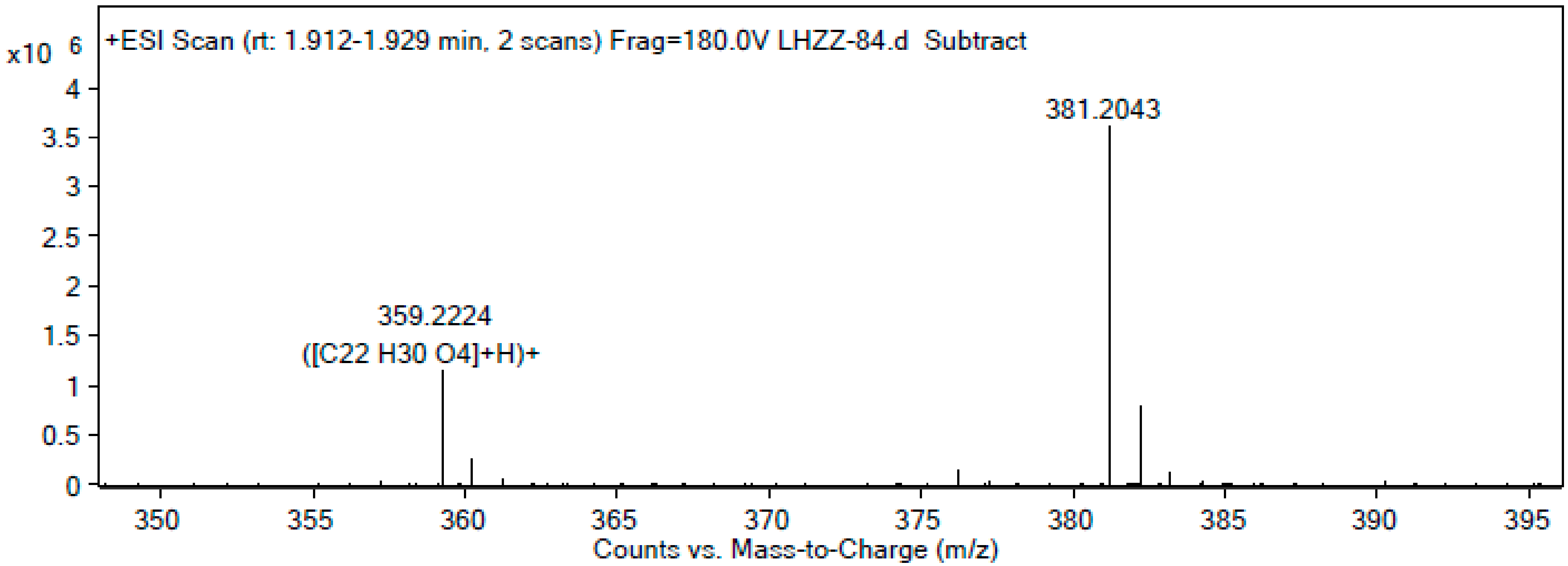

Figure S17: HR-ESI-MS spectrum of **2**.

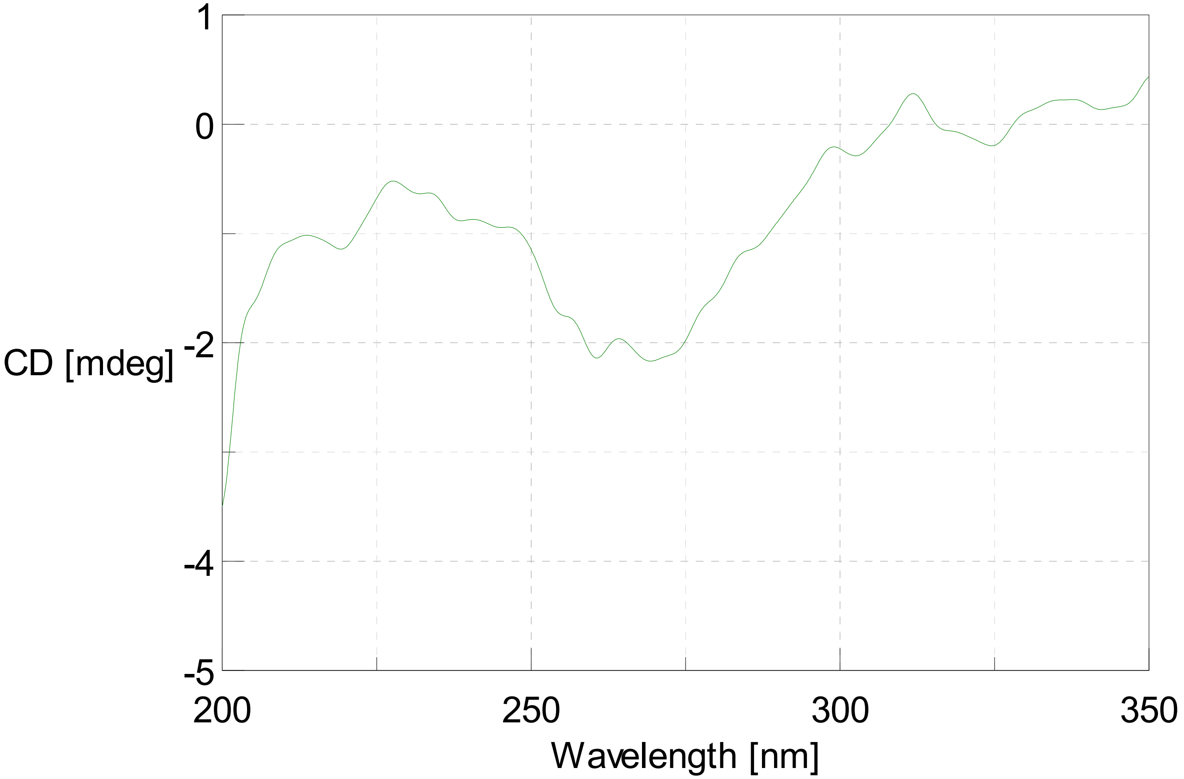

Figure S18: ECD sepctrum of **2**.

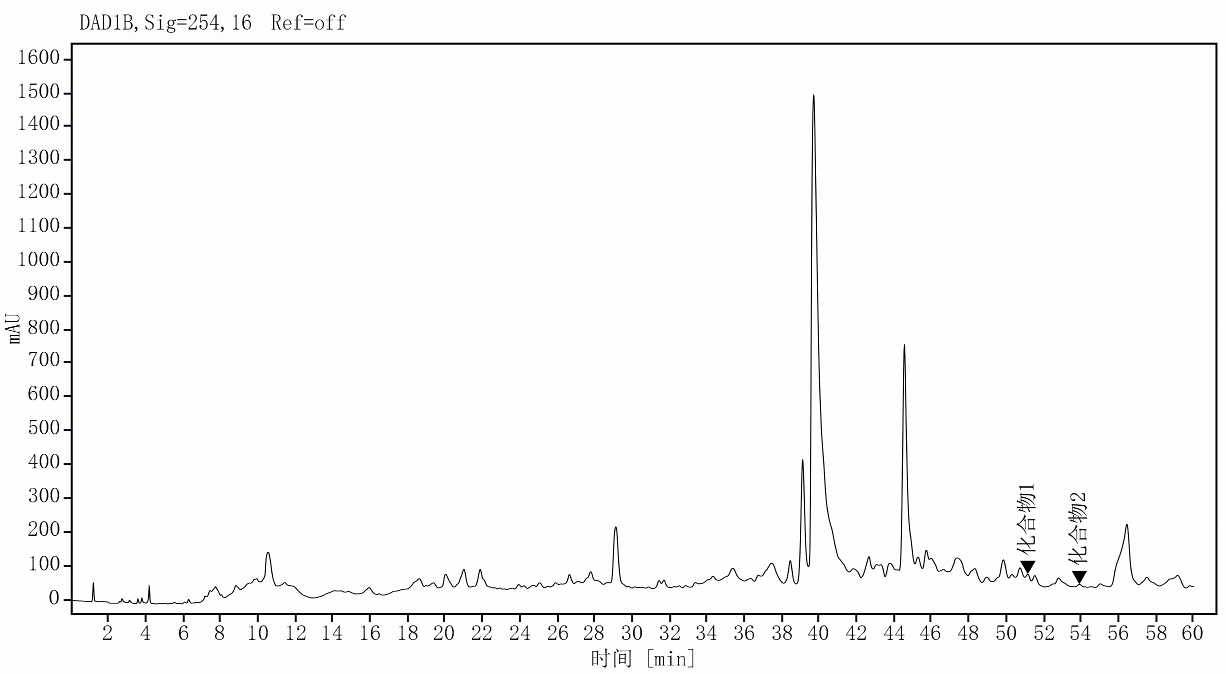

Figure S19: The HPLC chromatogram of the ethyl acetate extract.

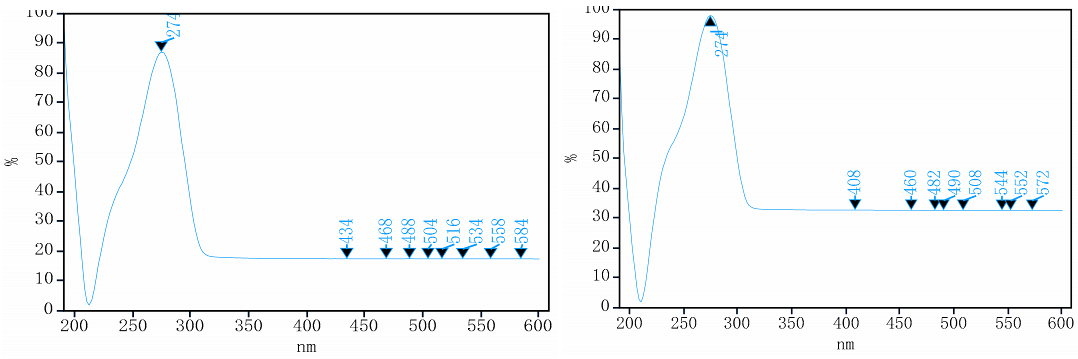

Figure S20: The corresponding UV spectrum of **1** and **2**.

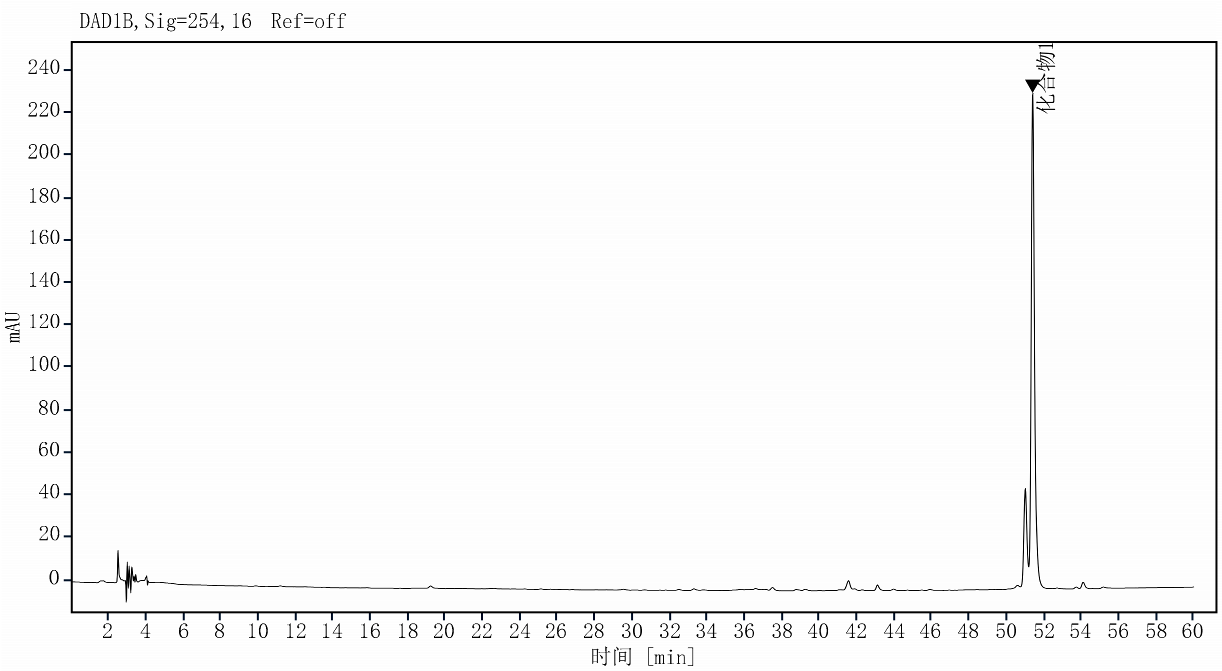

Figure S21: The HPLC chromatogram of **1**.

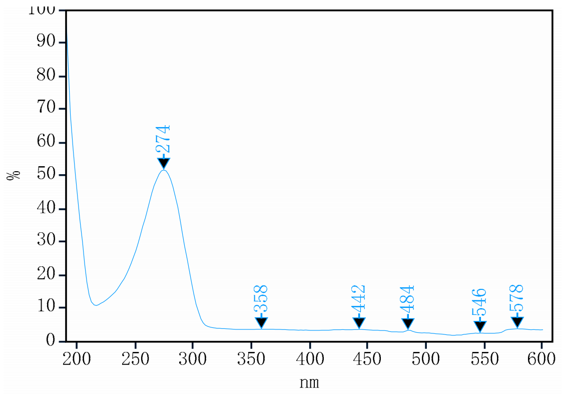

Figure S22: The corresponding UV spectrum of **1**.

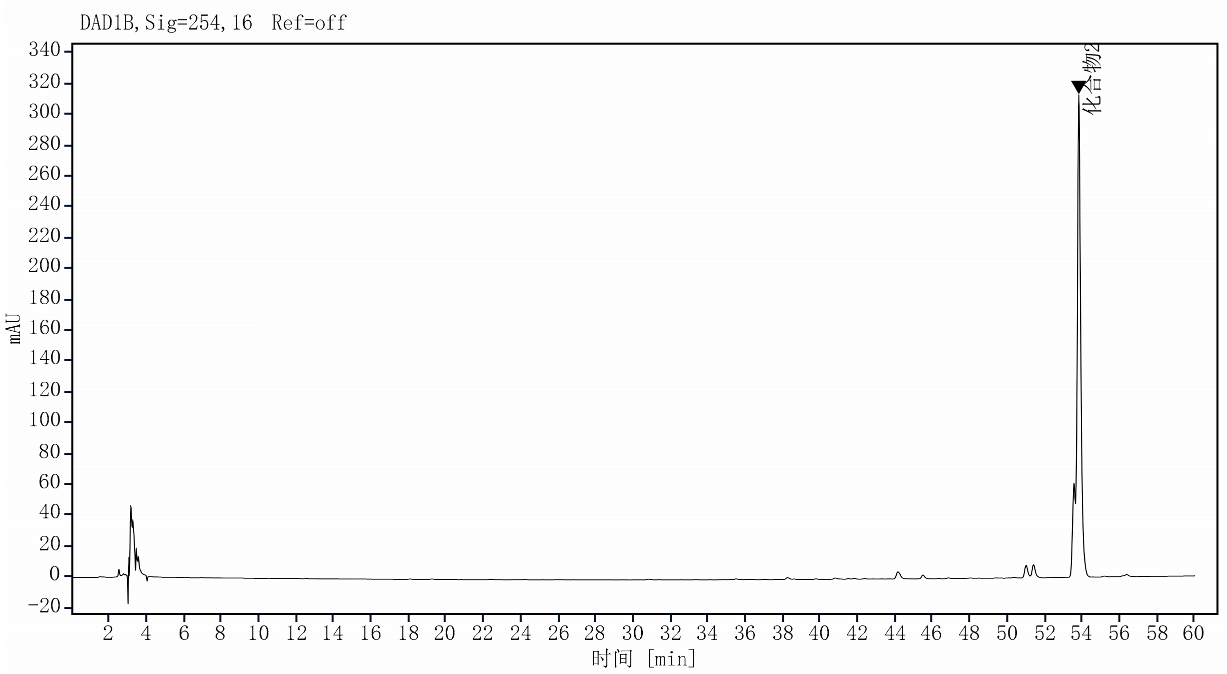

Figure S23: The HPLC chromatogram of **2**.

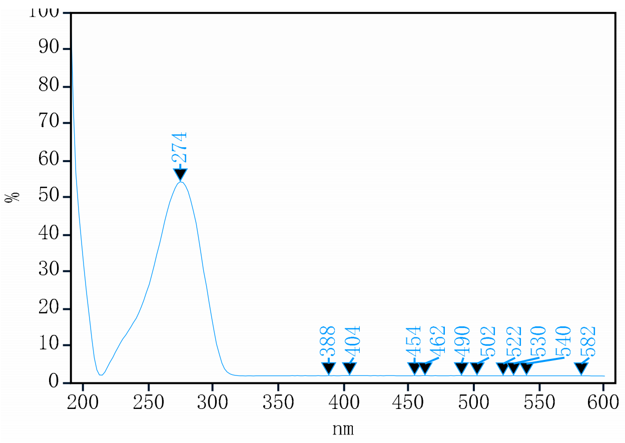

Figure S24: The corresponding UV spectrum of **2**.
